# SaeRS-Dependent Inhibition of Biofilm Formation in *Staphylococcus aureus* Newman

**DOI:** 10.1371/journal.pone.0123027

**Published:** 2015-04-08

**Authors:** David Cue, Jennifer M. Junecko, Mei G. Lei, Jon S. Blevins, Mark S. Smeltzer, Chia Y. Lee

**Affiliations:** Department of Microbiology and Immunology, University of Arkansas for Medical Sciences, Little Rock, AR, 72205, United States of America; Universitätsklinikum Hamburg-Eppendorf, GERMANY

## Abstract

The SaeRS two-component regulatory system of *Staphylococcus aureus* is known to affect the expression of many genes. The SaeS protein is the histidine kinase responsible for phosphorylation of the response regulator SaeR. In *S*. *aureus* Newman, the *sae* system is constitutively expressed due to a point mutation in *saeS*, relative to other *S*. *aureus* strains, which results in substitution of proline for leucine at amino acid 18. Strain Newman is unable to form a robust biofilm and we report here that the biofilm-deficient phenotype is due to the *saeS^P^* allele. Replacement of the Newman *saeS^P ^*with *saeS^L^*, or deletion of *saeRS*, resulted in a biofilm-proficient phenotype. Newman culture supernatants were observed to inhibit biofilm formation by other *S*. *aureus *strains, but did not affect biofilm formation by *S*. *epidermidis*. Culture supernatants of Newman *saeS^L ^*or Newman Δ*saeRS* had no significant effect on biofilm formation. The inhibitory factor was inactivated by incubation with proteinase K, but survived heating, indicating that the inhibitory protein is heat-stable. The inhibitory protein was found to affect the attachment step in biofilm formation, but had no effect on preformed biofilms. Replacement of *saeS^L^* with *saeS^P^* in the biofilm-proficient *S*. *aureus* USA300 FPR3757 resulted in the loss of biofilm formation. Culture supernatants of USA300 FPR3757 *saeS^P^*, did not inhibit biofilm formation by other staphylococci, suggesting that the inhibitory factor is produced but not secreted in the mutant strain. A number of biochemical methods were utilized to isolate the inhibitory protein. Although a number of candidate proteins were identified, none were found to be the actual inhibitor. In an effort to reduce the number of potential inhibitory genes, RNA-Seq analyses were done with wild-type strain Newman and the *saeS^L^* and Δ*saeRS* mutants. RNA-Seq results indicated that *sae* regulates many genes that may affect biofilm formation by Newman.

## Introduction

*Staphylococcus aureus* is a major nosocomial and community acquired pathogen causing a diverse array of infections ranging from superficial infections of the skin and mucosa to highly invasive and potentially lethal infections. Many bacterial infections are associated with biofilm formation. In the case of *S*. *aureus*, biofilms are associated with endocarditis, osteomyelitis, and infections associated with implanted medical devices such as catheters and prosthetic heart valves. Biofilm formation can serve to protect bacteria from antibiotics and host immune responses [1–4].

Bacterial biofilms are complex communities composed of layers of bacterial cells embedded within a glycoccalyx. Formation of biofilms occurs in distinct phases of attachment, accumulation and disassembly [1,4–7]. The attachment phase is often mediated by bacterial cell surface proteins, collectively known as MSCRAMMs, which bind to host plasma/extracellular matrix proteins such as fibronectin, fibrinogen and collagen [8]. Other bacterial proteins can bind directly to abiotic surfaces. The accumulation step in biofilm formation requires mechanisms for intercellular aggregation. Some staphylococci synthesize an exopolymer known as PIA, for polysaccharide intercellular adhesion that is composed of poly-N-acetyl glucosamine or PNAG. PNAG-containing biofilms often also contain bacterial proteins and extracellular DNA. The composition of staphylococcal biofilms is quite variable, with some strains being heavily dependent on PNAG and other strains being more dependent on extracellular proteins and DNA [4,6,9].

Biofilm formation is a highly regulated process, but what signals biofilm formation during infection is not understood [10–13]. Understanding the genetic regulation of biofilms is important, as bacterial regulatory factors represent targets for development of new antimicrobials. During the course of our work on biofilm formation by *S*. *aureus* strain Newman, we found that while Newman failed to form a robust biofilm, *sae* mutants of Newman could produce biofilms, thus implicating *sae* as a negative regulator of biofilm formation.

SaeRS was first identified from a transposon-insertion mutant deficient in exoprotein production and has subsequently been shown to control many *S*. *aureus* virulence determinants including surface proteins, toxins, and capsule biosynthesis components [13–17]. *saeRS* is transcribed as a 4-gene operon, *saePQRS*, with *saeS* encoding the sensor and *saeR* encoding the response regulator of a two-component regulatory system [18,19]. SaeP and SaeQ have been shown to interact with and regulate the activity of SaeS [20]. Following activation by environmental signals, SaeS is autophosphorylated, followed by phosphorylation of SaeR, which then binds to a specific target sequence to activate transcription of target genes [21,22]. Furthermore, SaeR binds the promoter region upstream of SaeP, creating a positive feedback loop [18]. SaeR must be phosphorylated to bind DNA and to function in vivo [21,23]. One sequenced *S*. *aureus* isolate (strain Newman) has an over-active SaeRS system due to a point mutation in *saeS* that substitutes a proline residue (SaeS^P^) for a leucine residue (SaeS^L^) [19]. This mutation results in a high level of SaeR phosphorylation leading to increased transcription of SaeR-regulated genes, including *saePQRS* [14,21,24,25]. While the *saeS* ^*P*^ allele is relatively rare, it was recently reported that the mutation is present in several uncharacterized strains (Gold Online Database project IDs 53133–53147).

Due to the importance of *sae* target genes, multiple researchers have determined the effects of repairing the point mutation in *saeS* ^*P*^ in strain Newman [12,14,15,24]. Although SaeRS is constitutively active in strain Newman, certain target genes are differentially regulated, designating two classifications of SaeRS targets. Class I target genes require the *saeS* ^*P*^ allele for expression, while Class II genes can be expressed by either the *saeS* ^*P*^ or the *saeS* ^*L*^ allele [24]. Differential expression of *sae*-regulated genes is believed to be due to the ratio of phosphorylated to unphosphorylated SaeR in cells, with the ratio being higher in strains with the *saeS* ^*P*^ allele [20,24]. Mainiero *et al*. [24] suggested that SaeS may possess a phosphatase activity that is defective in the SaeS^P^ protein. In support of this, Jeong *et al*. [20] found that SaeS^L^ does have phosphatase activity, which is activated by the interaction of SaeP and SaeQ with SaeS. The constitutive activity SaeS^P^ in Newman may be due to loss of catalytic activity or the inability of SaeS^P^ to interact with SaePQ. Either of these possibilities is consistent with the finding that SaeS^L^ is dominant over SaeS^P^, when *saeS* ^*L*^ is cloned into wild-type Newman [24]. Because SaeRS is activated by hydrogen peroxide and alpha-defensins and because many toxins are SaeRS-regulated, it has been hypothesized that this system could promote escape from PMNs after phagocytosis [22]. Indeed, a *saeRS* mutant strain demonstrated an impaired ability to survive in human neutrophils after phagocytosis [17]. We report here that strain Newman, with the *saeS* ^*P*^ allele, secretes a protein capable of inhibiting biofilm formation by other *S*. *aureus* strains. Moreover, deletion of *saeRS* ^*P*^, or replacement of SaeS^P^ with *saeS* ^*L*^, blocked production of the inhibitory protein by strain Newman. Additionally we performed RNA-Seq analysis to compare gene expression in strain Newman, Newman with a repaired *saeS* gene (*saeS* ^*L*^) and Newman deleted for *saeRS*.

## Results and Discussion

### A SaeRS-regulated protein prevents biofilm formation

**Fig 1:**
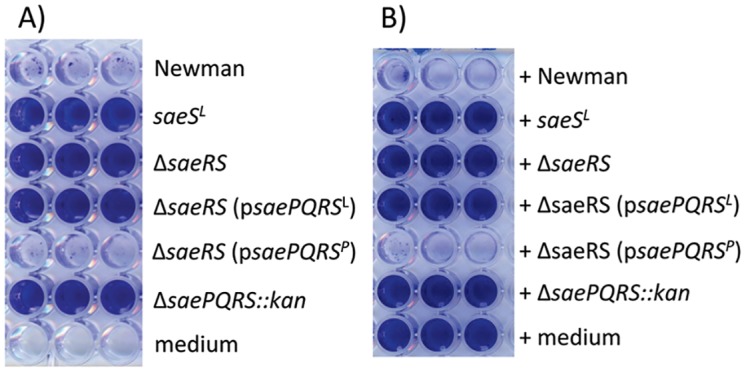
SaePQRS regulates biofilm formation by *S*. *aureus* strain Newman. (**A**) Stationary phase cultures of the indicated strains were diluted to an OD_660_ of 0.05 and inoculated into wells of a microtiter plate. The microtiter plate wells had been precoated with human plasma. After 16 h, biofilms were washed, fixed, and stained with crystal violet. The wells labeled “medium” indicates wells that contained sterile biofilm medium. (**B**) Inhibition of UAMS-1 biofilm formation by culture supernatants. Stationary phase culture supernatants of strain Newman and its derivatives were harvested, filter sterilized and added to microtiter plate wells preinoculated with *S*. *aureus* UAMS-1 suspended in biofilm medium. The source of each culture supernatant is listed to the right of the picture. The wells labeled “medium” indicates sterile TSB-0G medium was added in place of an exogenous culture supernatant. Plates were incubated and processed as described for A.

The capacity of *S*. *aureus* Newman to form a biofilm has been addressed by several laboratories. Some have reported that Newman can form a biofilm whereas others, including our laboratory, have concluded that strain Newman is a poor biofilm producer [12,15,26,27]. As stated above, strain Newman constitutively expresses the SaeRS two-component system. To determine if constitutive activation of SaeRS in Newman contributes to the biofilm-deficient phenotype, strains Newman, Newman *saeS* ^*L*^, Newman deleted for *sae* (Δ*saePQRS*::*kan* or *ΔsaeRS*) and the *ΔsaeRS* complemented with either *saePQRS* ^*P*^ or *saePQRS* ^*L*^, were assessed in a static microtiter plate biofilm assay. Strikingly, the *saeS* ^*L*^, Δ*saePQRS*::*kan* and *ΔsaeRS* strains formed robust biofilms compared to Newman wild-type (Fig 1A). Introduction of the *saePQRS* ^*P*^, but not *saePQRS* ^*L*^, bearing plasmid into the *ΔsaeRS* mutant resulted in restoration of the Newman wild-type, biofilm-deficient phenotype (Fig 1A).

We next tested the possibility that strain Newman may secrete a factor that inhibits biofilm formation. Supernatants from cultures of strain Newman and its derivatives were harvested and added to the biofilm medium at 25% (v/v). *S*. *aureus* strain UAMS-1 was subsequently inoculated into the medium, and biofilm assays were performed (Fig 1B). Supernatants from the *ΔsaeRS* or Δ*saePQRS*::*kan* strains did not inhibit biofilm formation by UAMS-1, while the supernatant from strain Newman did. In fact all of the biofilm forming derivatives of Newman were unable to inhibit biofilm formation by UAMS-1 whereas the biofilm deficient derivatives were able to inhibit (Fig 1B). These results demonstrated that Newman derivatives with the *saeS* ^*P*^ allele secrete a factor that inhibits biofilm formation.

**Fig 2:**
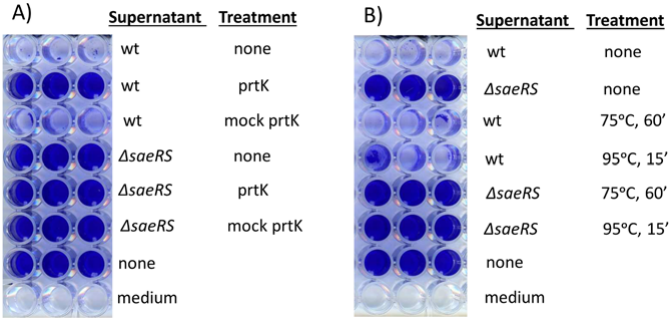
The biofilm inhibitory factor is a heat stable protein. Stationary phase culture supernatants of strain Newman (wt) and Newman *ΔsaeRS* were harvested, filter sterilized, and diluted into biofilm medium. Anti-biofilm activity of supernatants was tested against *S*. *aureus* strain UAMS-1. (**A**) Proteinase K treatment of culture supernatants. Supernatants from the indicated strains were incubated with proteinase K and Tris buffer, or buffer only (mock prtK), at 42°C for 1 hr and then 95°C for 10 minutes, before adding to biofilm medium. “None” indicates wells containing UAMS-1 but no culture supernatant. Wells labeled “medium” contained sterile biofilm medium. (**B**) Supernatants were incubated at the indicated temperatures for the indicated time periods before adding to wells containing UAMS-1 suspended in biofilm medium.

To determine if the inhibitor is a protein, supernatants were treated with proteinase K (New England Biolabs) and the proteinase was subsequently heat inactivated. As shown in Fig 2A, proteinase K-treated supernatant from strain Newman did not inhibit biofilm formation by strain UAMS-1, suggesting the inhibitory factor is a protein. To further characterize the protein, supernatants were subjected to heating at 75°C for 60 min or 95°C for 15 minutes. Heat treated supernatants were then added to the biofilm media at 25% and strain UAMS-1 was used for the biofilm assay. None of the treatments inactivated the protein (Fig 2B), suggesting that the protein is heat stable.

### Regulation of attachment and dispersal by SaeRS

**Fig 3:**
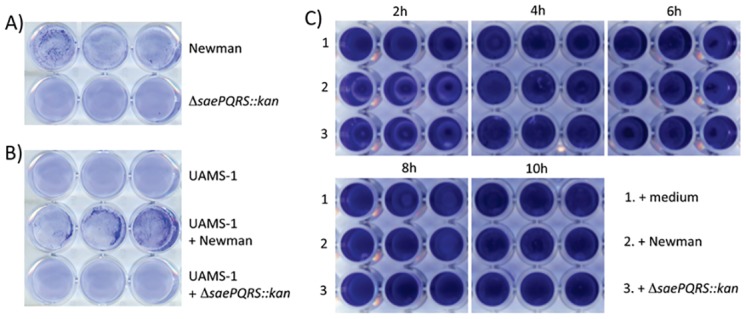
*saeRS* regulates attachment but not dispersal. (**A**) Stationary phase cultures of Newman and the Δ*saePQRS*::*kan* strain were diluted into biofilm medium, inoculated into a 24-well plate and allowed to attach for 1 h at 37°C before washing and staining with crystal violet. (**B**) Supernatants from Newman and Newman Δ*saePQRS*::*kan* were diluted into biofilm media and the attachment phenotype of strain UAMS-1 was tested with and without supernatant supplementation. (**C**) Newman (row 2) and Newman Δ*saePQRS*::*kan* (row 3) culture supernatants were added at the indicated times following inoculation with UAMS-1. Wells in row 1 were supplemented with sterile medium rather than culture supernatant.

To test whether the inhibitory protein affects the attachment step of biofilm formation, attachment assays were performed by diluting mid-log phase bacteria into biofilm media and inoculating plasma-coated 12-well plates. Bacteria were incubated for 1 h at 37°C, then were washed, fixed, and stained with crystal violet. Newman wild-type attached as distinct clumps, while the Δ*saePQRS*::*kan* strain attached as a uniform layer of cells (Fig 3A). To confirm this effect, a strain UAMS-1 culture was supplemented with supernatants from either strain Newman or Newman Δ*saePQRS*::*kan* and inoculated as stated above. Strain UAMS-1 cultures supplemented with supernatant from strain Newman exhibited the same clumping phenotype as strain Newman, whereas UAMS-1 incubated with the Δ*saePQRS*::*kan* mutant supernatant attached as a uniform layer of cells (Fig 3B). These results suggested the SaeRS-regulated protein affects the attachment step of biofilm formation. To test a possible role of SaeRS in biofilm dispersal, strain UAMS-1 was inoculated into a plasma-coated microtiter plate at time 0. Every two hours for 10 hours post inoculation of strain UAMS-1, the media was changed to one of the following: 1) fresh biofilm media, 2) fresh biofilm media supplemented with Newman supernatant, or 3) fresh biofilm media supplemented with the Δ*saePQRS*::*kan m*utant supernatant. At 24 h post inoculation, biofilms were washed, fixed, and stained with crystal violet. Media supplemented with strain Newman supernatant did not affect mature biofilms (Fig 3C), suggesting that the putative inhibitory protein does not have the capacity to disrupt a mature biofilm. Collectively, these data suggest that a SaeRS^P^-regulated protein inhibits the attachment step in biofilm formation.

### Strain-dependent expression of the biofilm inhibitory protein

**Fig 4:**
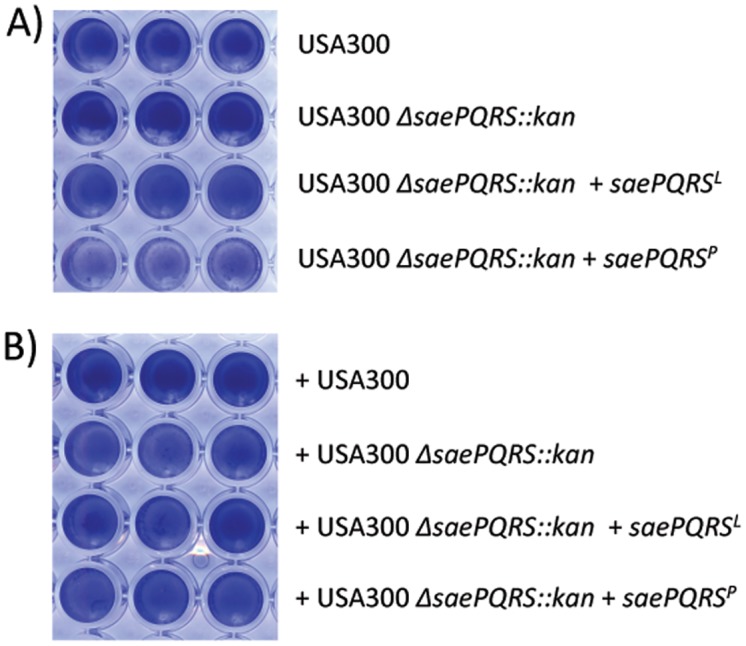
Strain-dependent production of the biofilm inhibitory protein. (**A**) Biofilm formation by strain USA300 FPR 3757 and derivatives. Wells were inoculated with the indicated USA 300 FBR 3757 derivatives. +*saePQRS* ^*L*^ and *+saePQRS* ^*P*^ indicate the presence of plasmids pCWSAE50 or pCWSAE51, respectively. (**B**) Anti-biofilm activity of USA300 derivatives. Culture supernatants from the strains listed to the right of the figure were tested for inhibition of biofilm formation by strain UAMS-1.

As stated previously, strain Newman has a point mutation in *saeS* that results in constitutive activation of the SaeRS two-component system. Our data suggested that a consequence of this constitutive activation was the inability to form a biofilm. To determine if the *saeS* ^*P*^ allele would affect other *S*. *aureus* strains, a SaeRS constitutively active strain was constructed in the USA300 FPR 3757 (USA300) background. First, the Δ*saePQRS*::*kan* mutation was introduced into wild-type strain USA300 FPR 3757 via phage transduction. Next, either the *saePQRS* operon from strain Newman (*saePQRS* ^*P*^) or from strain 8325–4 (*saePQRS* ^*L*^) was introduced into USA300 Δ*saePQRS*::*kan*, using pCWSAE51 or pCWSAE50, respectively. The vector carrying the *sae* operons is a single copy integration vector that integrates within the *hlb* gene at the phage Ø13 attachment site [24]. The benefit of using an integration vector is stable maintenance of the construct in the chromosome allowing biological assays to be performed without the use of antibiotics to maintain the plasmid. Resulting strains were tested for biofilm production and culture supernatants from planktonic cultures were subsequently tested for anti-biofilm activity against strain UAMS-1. As shown in Fig 4A, wild-type strain USA300 and the Δ*saePQRS*::*kan* mutant formed a biofilm, and complementation of Δ*saePQRS*::*kan* with *saePQRS* from strain 8325–4 (denoted *saePQRS* ^*L*^) resulted in no change in the biofilm phenotype. Interestingly, complementation of Δ*saePQRS*::*kan* with *saePQRS* from strain Newman (denoted *saePQRS* ^*P*^) resulted in an apparent reduction in biofilm formation, further confirming our hypothesis that constitutive activation of SaeRS limits biofilm formation. To assess production of the secreted biofilm inhibitory protein, USA300 and derivatives were grown to stationary phase and culture supernatants were harvested, filter sterilized and added to biofilm assays of strain UAMS-1. Culture supernatants from strain USA300 expressing *saePQRS* ^*P*^ did not contain any apparent anti-biofilm activity (Fig 4B), which suggests the inhibitory factor is not secreted by USA300. It is possible that the inhibitory protein is associated with the cell surface in USA300, but is released into the environment by Newman. Newman does encode some truncated versions of cell-wall anchored proteins that, due to the truncation, would no longer be anchored [25,28]. Identification of the inhibitory protein should aid in understanding this result. It is worth noting that another USA300 isolate (USA300 LAC) does secrete a DNA nuclease that can inhibit biofilm of other staphylococci [29]. However, as discussed below, the nuclease is distinct from the inhibitory factor produced by Newman.

### Inhibition of biofilm formation by other *S*. *aureus* and *S*. *epidermidis* isolates

**Fig 5:**
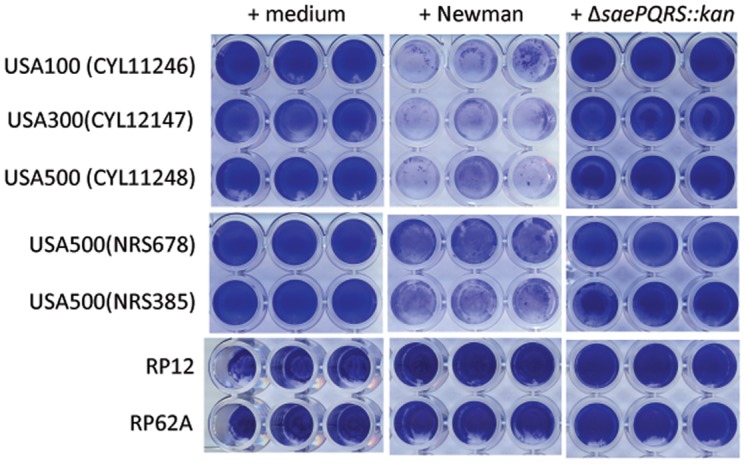
The biofilm inhibitory protein limits biofilm in *S*. *aureus* but not *S*. *epidermidis* strains. Culture supernatants from strain Newman or the Δ*saePQRS*::*kan* mutant were added to biofilm medium and anti-biofilm activity was tested against the bacterial strains listed to the left of the figure. “Medium” indicates that sterile medium was added in place of culture supernatant.

The results above indicate that a secreted protein produced by strain Newman inhibited biofilm formation by strain UAMS-1 when added to biofilm medium at the time of inoculation. To test anti-biofilm activity of the putative secreted protein against other staphylococci, including *S*. *aureus* strains USA100, USA300, three isolates of USA500, and *S*. *epidermidis* strains RP12 and RP62A were inoculated into biofilm media containing supernatant from strain Newman. The putative protein effectively inhibited biofilm formation by all *S*. *aureus* strains tested but did not inhibit biofilm formation by either *S*. *epidermidis* strain. Supernatant from Newman Δ*saePQRS*::*kan* did not affect biofilm formation by any strain (Fig 5). The lack of an inhibitory effect on *S*. *epidermidis* could be due to species-dependent differences in biofilm formation. *S*. *epidermidis* relies more heavily on exopolysaccharides than exoproteins for biofilm formation. Some *S*. *aureus* strains on the other hand, rely primarily on exoproteins for biofilm formation, at least in the presence of plasma proteins [4,6]. Alternatively, the actual target of the inhibitor may not be expressed by *S*. *epidermidis*.

### Screens to identify the biofilm inhibitor

A variety of biochemical methods were used in attempts to identify the biofilm inhibitory factor produced by strain Newman. These included fractionation of culture supernatants using Ultra centrifugal filters, NH_4_SO_4_ precipitation, size exclusion and ion exchange chromatography and gel electrophoresis or a combination of these methods. Using these methods a number of candidate proteins were identified and these were subjected to trypsin digestion and tandem mass spectrometry sequencing and identification. S1 Table lists the candidate genes identified as possibly encoding the inhibitor protein. Each of the candidate proteins listed in S1 Table was tested for anti-biofilm activity using several genetic approaches, including insertional inactivation, gene deletion, and complementation. However, despite our efforts, the inhibitory protein was not identified.

In summary, our studies show that strain Newman expresses a heat stable extracellular protein, approximately 50 kDa in size, that can inhibit biofilm formation by the producing organism, as well as other strains of *S*. *aureus*. The protein is regulated by *saePQRS* and is dependent on the presence of *saeS* ^*P*^ allele for an effective level of expression. Preparations of the inhibitory factor seem to have high activity, suggesting the inhibitor is an enzyme or a signaling factor. Although we tested a number of candidate genes, the gene encoding the inhibitor was not identified. It is possible that the inhibitory factor is actually more than one protein and inactivation of a single gene is insufficient for complete loss of activity.

### RNA-Seq analysis of the SaeRS transcriptome

Based on the data summarized above, we anticipated that the gene encoding the biofilm inhibitory factor would be expressed at a higher level in strain Newman than in either the *saeS* ^*L*^ or *Δsae* strains. In an effort to identify the Newman encoded biofilm inhibitory protein, we carried out RNA-seq analysis using RNA from CYL5876 (Newman SaeRS^P^), CYL11481 (Newman saeS^L^), and CYL11771 (Newman *ΔsaeRS*). Although several previous studies have identified SaeRS-regulated genes [17,24,30,31], no studies to date have compared three strains with varying levels of SaeRS activity. Furthermore, previous transcriptional profiling studies employed microarray-based approaches. Microarrays are based on annotated genes and will not detect every transcriptionally active region in the genome of all *S*. *aureus* strains. RNA-Seq does not rely on gene probes and directly sequences and identifies all transcriptionally active regions in a genome rather than detecting only annotated genes [32,33]. Our data confirm previous reports that SaeRS regulates many genes, and also confirmed previous findings regarding strain-dependent differences in SaeRS activity. As expected, our analysis is more comprehensive and identified far more genes in the SaeRS region than earlier reports [17,24,30,31].

The RNA–Seq results indicated that expression of 225 genes was at least 2-fold lower in the *ΔsaeRS* strain than in strain Newman (S2 Table) and 80 genes were expressed at a lower level in the *ΔsaeRS* strain than by *saeS* ^*L*^ strain (S3 Table). Expression of 80 genes was lower in the *saeS* ^*L*^ strain relative to the wild-type (S4 Table). These genes represent those that are under positive regulation by *sae*. A smaller set of genes, 64, are up regulated in the *ΔsaeRS* strain relative to wild type Newman (S5 Table). Many of the latter group are up regulated in the SaeRS^L^ strain as well, thus these genes are negatively regulated by *sae* (S6 and S7 Tables). It seems probable that the gene(s) encoding the biofilm inhibitory protein would be listed in both S2 and S4 Tables.

It has been reported that SaeR must be phosphorylated to bind DNA and that a mutation of the phosphorylation site in SaeR results in a complete loss of *sae* activity [21,24]. Mainiero *et al*. (2010) [24] argued that transcription of some *saeRS*-regulated genes requires a high level of SaeR phosphorylation whereas transcription of other genes can occur with a lower level of SaeR phosphorylation. They also proposed that low level phosphorylation of SaeR can occur in the absence of SaeS. The constitutive activity of SaeS^P^ results in a high level of SaeS kinase activity, or possibly a low level of phosphatase activity, resulting in a high ratio of phosphorylated to unphosphorylated SaeR [18,20,24]. Our RNA-Seq results identified a number of genes where there is an apparent dose-dependent response to SaeRS levels (S8 Table), meaning that expression of a particular gene is highest in strain Newman (with constitutively active SaeRS) and lowest in the Δ*saeRS* strain. Expression of these genes is intermediate in the *saeS* ^*L*^ strain. It seems likely that these results reflect different levels of phosphorylated SaeR in the three strains. There are a number of genes that appear to be expressed nearly equally well in the wild type and the *saeS* ^*L*^ strains, but poorly expressed in the deletion strain. The most striking example of this is the *hla* gene (NWMN_1073) which is expressed at a much higher level in the *saeS* ^*P*^ and *saeS* ^*L*^ strains than in the deletion strain. (S2 and S3 Tables) The *saeS* ^*L*^ strain apparently has a level of phosphorylated SaeR sufficient for full expression of these genes.

### Confirmation of RNA-Seq by real-time RT-PCR

**Fig 6:**
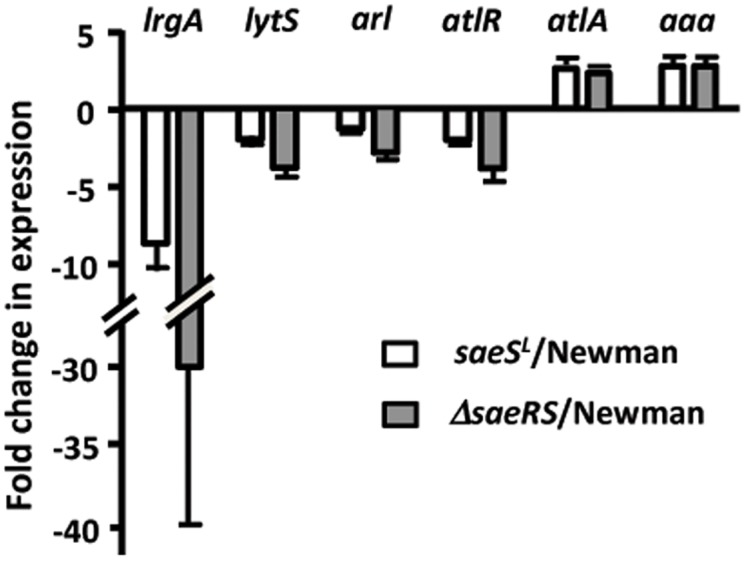
Confirmation of RNA-Seq data using RT-qPCR. RNA was isolated from cultures of strain Newman (*saeS* ^*P*^), CYL11481 (*saeS* ^L^) and CYL11771 (Δ*saeRS*) for use in RT-qPCR assays. Values for the *saeS* ^L^ (open bars) and Δ*saeRS* (grey bars) strains are expressed relative to wild type Newman, which was assigned an arbitrary value of 1. All assays were performed with at least two RNA preparations obtained from separate cultures.

To confirm the RNA-Seq results, we performed real-time RT-PCR of some select genes. Cultures of strains CYL5876, CYL11481, and CYL11771 were grown as for the RNA-Seq analysis. RNA was isolated from these cultures as described for RNA-Seq analysis except that rRNA depletion was not performed. As we are interested in genes that might influence biofilm formation, we decided to focus on genes predicted to affect the level of extracellular DNA (eDNA). We also verified expression of saeR in the three test strains (*saeR* in the *saeS* ^*L*^ strain was 4.2±1.2% of the wild type and undetectable in the *saeRS* strain). We measured expression of 6 *saeRS*-regulated genes, *lytS*, *lrgA*, *atlR*, *atlA*, *arlR* and *aaa*, that could potentially affect autolysis and eDNA release by strain Newman. The results shown in Fig 6 are in general agreement with the RNA-Seq results, although differences in gene expression between the strains were generally greater in the RNA-Seq analysis. As anticipated, expression levels of *lytS*, *lrgA*, *arlR* and *atlR* were highest in Newman and lowest in the deletion strain with expression in the *saeS* ^*L*^ strain being intermediate. The reverse pattern was observed for *atlA* and *aaa*; where the expression levels were greatest in Δ*saeRS*≥*saeS* ^L^>Newman. Most of these genes are probably indirectly regulated by *saeRS*. For example, *lytS* and *atlR* regulate expression of *lrgAB* and *atlA*, respectively, but neither *lytS* nor *atlR* has a predicted SaeR binding site in their promoter region [34]. The *aaa* gene does not appear to have a SaeR binding site either and may not be directly regulated by *sae*. SaeR has been shown to bind to the *arlR* promoter, suggesting direct regulation of this gene by *saeRS*. The *arlRS* 2-component system is involved in the negative regulation of the LytN autolysin [35] but an effect of *sae* on *lytN* was not apparent in our analysis.

### Regulation of autolysis and eDNA accumulation by *saeRS*

Autolysis could result in the release of bacterial DNA into the environment and extracellular or eDNA has been proposed to play a positive role in biofilm formation, therefore, we were interested in how *sae* would affect cell lysis and release of bacterial DNA. LytSR is a 2-component regulatory system that activates expression of *lrgAB* [34]. LrgA is an antiholin and an antagonist of CidA, a holin-like protein that has been proposed to disrupt the cell membrane leading to cell lysis [36–38]. Because *saeS* ^*P*^ upregulates *lytSR* and *lrgAB*, *saeS* ^*P*^ would be predicted to decrease the release of cellular DNA into the environment, relative to the *saeS* ^*L*^ and *ΔsaeRS* strains [37]. Thus the overexpression of *lytS* in wild-type Newman would have a negative impact on biofilm formation. The impact of *sae* on biofilm would be lessened in strains that express the *saeS* ^*L*^ allele. The negative effect of *saeS* ^*P*^ on expression of *atlA*, which encodes the major *S*. *aureus* autolysin, would also be predicted to decrease autolysis and DNA release [39,40]. In this instance, however, the inhibitory effect would be manifest through *sae* upregulation of *atlR* which encodes a repressor of *altA* [40]. It is important to note that *atlA* has been shown to increase biofilm in *S*. *aureus* by promoting attachment and early biofilm formation [40,41]. Increased SaeRS does decrease expression of *aaa*, which encodes a N-acetylmuramoyl-L-alanine amidase precursor [42]. The amidase may well contribute to the cell lysis. Phages ØNM1 and ØNM4 encode a holin (NWMN_1770) and an amidase (NWMN_0313), respectively. However, these genes are increased slightly in the *saeS* ^*L*^ but not in the Δ*saeRS* strain.

**Fig 7:**
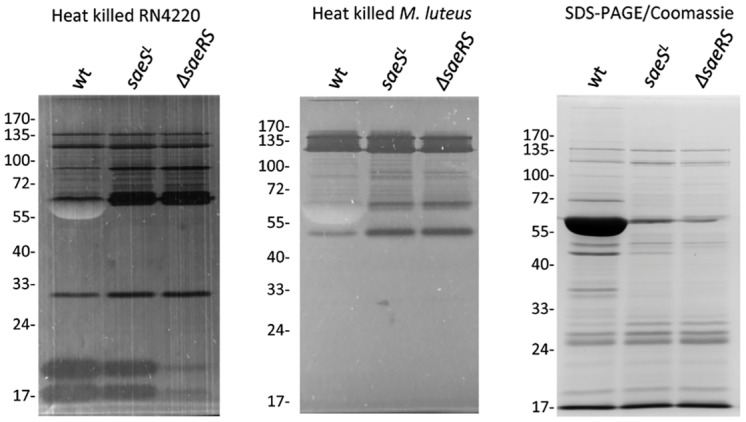
Zymograms of *S*. *aureus* murein hydrolase activity. Cell free extracts of strains Newman (wt), *saeS* ^*L*^ (CYL11481) and Δ*saeRS* (CYL11771) were run on 10% acrylamide-SDS gels containing heat killed *S*. *aureus* RN4220 cells (leftmost panel) or heat killed *Micrococcus luteus* cells (middle panel). Numbers to the left of each gel indicate the molecular weights of size standards. Dark bands indicate regions of murein hydrolase activity. The large clear band migrating at approximately 60 kDa is presumably the Map protein, which is highly expressed in strain Newman. The rightmost panel is a Coomassie Blue-stained gel showing the same extracts used for the other panels. The large band migrating at approximately 60 kDa is presumably the Map protein.

The transcriptional analysis of murein hydrolase genes summarized above, suggested that the Δ*saeRS* and *saeS* ^*L*^ strains may produce higher levels of these enzymes than does wild type Newman. To test this possibility, zymographic analyses of the three strains were performed (Fig 7). Extracts of these strains were prepared and fractionated on gels containing either heat-killed *S*. *aureus* strain RN4220 cells or cells of *Micrococcus luteus*. As shown in Fig 7, increased murein hydrolase activity, relative to Newman, was associated with both the *saeS* ^*L*^ and Δ*saeRS* strains. The differences were most prominent for protein bands of approximately 60 and 90 KDa, and somewhat less prominent for a protein migrating at approximately 30 kDa. Additionally, the Δ*saeRS* strain was deficient in expression of 2 small hydrolases that migrated at the 17 to 20 kDa range.

The *atlA* encoded protein is a bifunctional N-acetyl-glucosaminindase (GL) and N-acetylmuramyl-L-alanine amidase (AM), expressed as a 137 kDa precursor that is processed to 53.6 kDa and 63 kDa AM proteins [41]. It seems likely that the approximate 60 kDa protein in the RN4220 zymogram (Fig 7, leftmost panel) represents the AtlA protein. The *aaa* gene encodes a 35.8 kDa precursor AM [42]. It is possible that the approximate 30 kDa hydrolase, which is expressed at a somewhat higher level in the Δ*saeRS* and *saeS* ^*L*^ strains, represents the *aaa* gene product. The LytM and LytH autolysins are of approximately the same size, but neither appeared to be regulated by *sae*.

Increased expression of at least 2 autolysins is apparent in the *M*. *luteus* zymogram (Fig 7, middle panel). Both the *saeRS* and *saeS* ^*L*^ strains show increased expression of 50 and 68 kDa enzymes. These could represent the AtlA GL and AM enzymes, respectively. While we have not verified the identities of the various autolysins, these results do verify that constitutive expression of *saeRS* in Newman does decrease autolytic activity associated with *S*. *aureus* cells.

**Fig 8:**
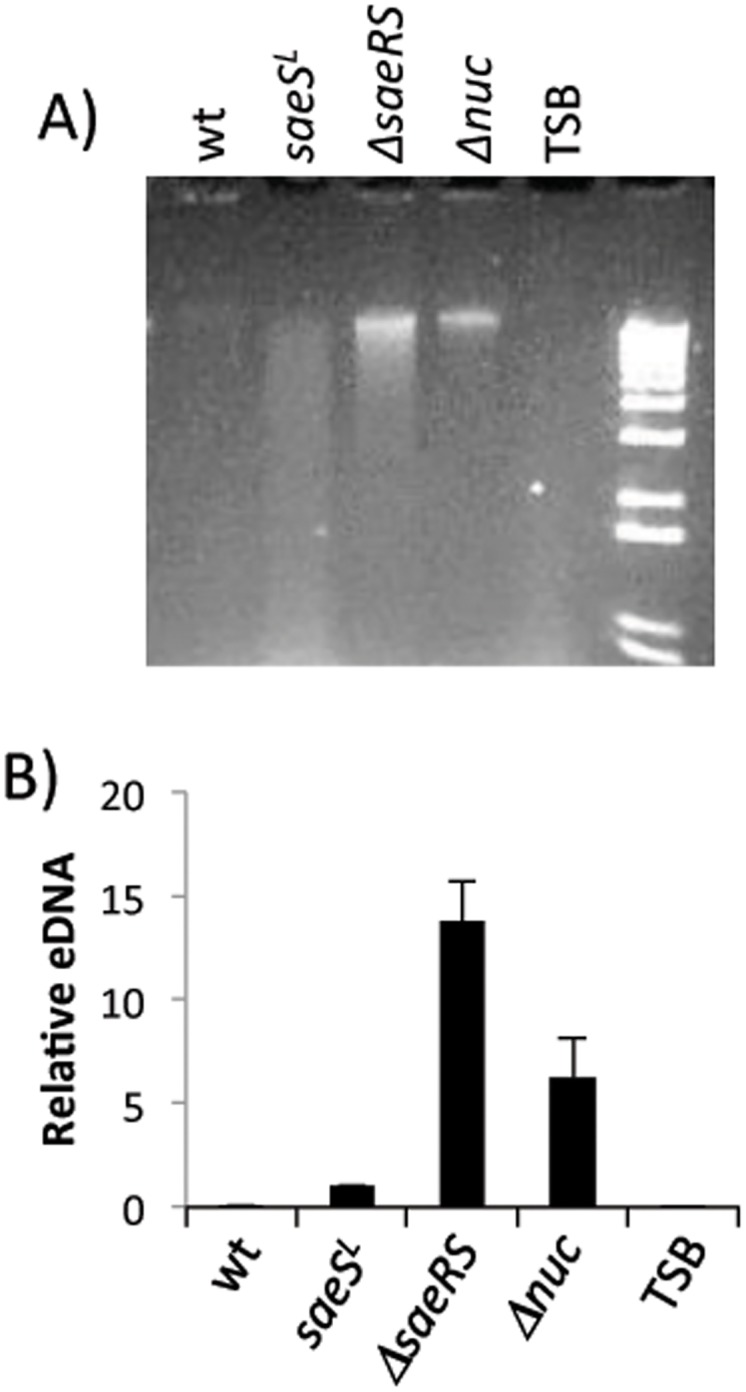
Extracellular (e) DNA in culture supernatants. Culture supernatants from strain Newman and its derivatives were filter sterilized, extracted with phenol-chloroform and ethanol precipitated. Precipitates were suspended in H_2_O. The volumes of water were varied to compensate for slight differences in OD_660_ of the initial cultures. (**A**) Agarose gel of isolated eDNAs. (**B**) qPCR results. qPCR was performed with isolated eDNAs using oligonucleotide primers specific for the *hu* gene. Data are expressed relative to the amount of eDNA in the *saeS* ^L^ strain supernatant, which was arbitrarily assigned a value of 1. Data are means from two independent cultures of each strain. *Δnuc* indicates a nuclease deficient mutant of Newman. TSB indicates material isolated from sterile culture medium (TSB-0G).

In order to determine if SaeRS does impact eDNA accumulation, we quantified the amount of DNA present in culture supernatants (Fig 8). A mutant (*Δnuc*) strain that does not express the secreted nuclease was included in our analysis as a positive control for eDNA accumulation. The amount of eDNA was over 10-fold higher in cultures of the *ΔsaeRS* strain than the *saeS* ^*L*^ strain and eDNA was essentially undetectable for wild type Newman. These results are consistent with the proposal that *saeS* ^*P*^ is a negative regulator of autolysis and eDNA production. The fact that eDNA was higher in the *ΔsaeRS* strain than in the Δ*nuc* strain suggests that factors other than nuclease affect eDNA accumulation.

The positive activation of *nuc* and *lrgAB* and the negative repression of autolysis genes by SaeRS^P^, would suggest that eDNA degradation may play a role in the biofilm deficient phenotype of Newman. Additionally, Nuc is a thermostable extracellular protein which is highly expressed in Newman. Nuc was previously shown to inhibit biofilm formation by *S*. *aureus* USA300 LAC and culture supernatants of USA300 LAC were found to inhibit biofilm formation by other strains [29]. Therefore, it seemed likely that Nuc could be the biofilm inhibitor produced by Newman. Indeed, Nuc was identified during our earlier screening by biochemical methods (S1 Table). However, Newman *nuc*::*ltrB* was still biofilm negative (S1 Fig), indicating that it is not the inhibitory factor that we were looking for. To test whether *lrgAB* plays a role in biofilm formation in Newman, a *lrgAB*::*erm* single mutant of Newman was generated and tested. The mutation did not affect the biofilm negative phenotype of Newman (S1 Fig). These results suggest that neither *nuc* nor *lrgAB* are involved in biofilm formation in Newman. Although Newman produces a significant amount of nuclease (Fig 7) any inhibitory effect appears to be masked by the yet to be identified inhibitory protein produced by Newman.

### Regulation of bacteriophage genes by *saeRS*

Strain Newman is known to carry 4 prophages, ØNM1, ØNM2, ØNM3 and ØNM4 that contribute to pathogenesis [25,43,44]. We found that each of these prophages has genes that are subject to *sae* regulation (S9 Table). ØNM3 is a defective prophage that encodes multiple virulence factors including *sea* (enterotoxin A), *sak* (staphylokinase), *chp* (chemotaxis-inhibitor protein), and *scn* (staphylococcal complement inhibitor). Of these, *sak*, *chp* and *scn* have predicted SaeR binding sites in their promoter regions [21] and the RNA-Seq analysis indicated all are regulated by *sae*. RNA-Seq also revealed that at least 15 other ØNM3 genes are regulated by *sae* including genes encoding head proteins and DNA replication and transcription factors. Because ØNM3 is defective, it seems unlikely that the prophage would excise from the chromosome and undergo replication. It is possible that ØNM3 may function as a helper phage for other bacteriophages or for packaging and transfer of other *S*. *aureus* mobile genetic elements. The ØNM3 genome does encode an HNH endonuclease of the type reported to be involved in phage-mediated horizontal gene transfer in *S*. *aureus* and other bacteria [45]. Our results indicate that *saeRS* could possibly play a role in horizontal gene transfer in *S*. *aureus* via the activation of prophage genes.

Interestingly, numerous ØNM4 genes are expressed at a higher level in strain Newman than in the *saeS* ^*L*^ strain. These genes seem to represent most of the NM4 genome including genes involved in prohead and tail assembly, DNA packaging and host cell lysis [43]. It may be that the ØNM4 prophage is induced to undergo lytic growth at a higher frequency in Newman than in the *saeS* ^*L*^ strain. The differences in expression level are small, but could well be significant as several ØNM4 genes are known to contribute to pathogenesis [43,44]. It is anticipated that prophage induction would ultimately result in lysis of bacterial cells and release of cell constituents into the environment.

To determine if the biofilm inhibitory protein might be encoded and/or regulated by a strain Newman prophage, we tested *S*. *aureus* strain TB3 for biofilm formation. Strain TB3 is a derivative of Newman which has been cured of prophages NM1, NM2, and NM4 [43]. We also tested strain TB1(pCWsae51). Strain TB1 is an NM3-deleted strain but also has a mutation in the *sae* locus (T. Bae, personal communication). We therefore constructed TB1(pCWsae51) to provide a complemented copy of *saeS* ^*P*^. None of the prophage deleted strains produced a biofilm (S2 Fig) suggesting that the *sae* regulated biofilm inhibitor is not encoded by any of the Newman prophage.

### Proteases are not involved in biofilm inhibition

It has been reported that proteases play a key role in biofilm inhibition and the transcriptional regulator, SarA, is the major repressor of proteinase production. Ten proteases were recognized in *S*. *aureus*. In strain Newman, SaeRS act synergistically with SarA to repress proteases, but the SaeRS effect is less than SarA [12]. Two proteinases, SspB and Aur were found in Newman culture supernatants, but mutations of the *sspB* or *aur* genes did not affect biofilm inhibition (S1 Table). In our RNA-seq analysis, we also found that the six serine proteinases encoded by the *splABCDEF* operon were under positive regulation by *saeRS* (S2 Table), thus it seemed possible that one or more of the *spl*-encoded enzymes could be the biofilm inhibitory factor. However, deletion of the *spl* operon had no effect on biofilm (S3 Fig). Since the other two proteases were not affected by SaeRS, these results indicate that the inhibitor *per se* is unlikely a protease or a group of proteases.

Because SaeS^P^ is a negative regulator of extracellular proteinases [12,15] (S2 Table), it seemed possible that the putative inhibitory protein may be degraded by a proteinase in the *saePQRS*::*kan* strain such that the supernatant is no longer capable of inhibiting biofilm formation. To test this possibility, experiments were performed using mixed culture supernatants following the rationale that a proteinase in the *saePQRS*::*kan* mutant could degrade the inhibitory protein in wild-type strain Newman supernatants. Supernatants were combined in ratios of Newman: *saePQRS*::*kan* at 4:1, 1:1 and 1:4 and incubated overnight at 37°C (S4 Fig). Supernatant mixtures were then TCA precipitated and protein pellets re-suspended in the initial volume of strain Newman supernatant. Samples were dialyzed overnight to remove residual TCA and tested for anti-biofilm activity against strain UAMS-1. Every sample that contained strain Newman supernatant exhibited anti-biofilm activity, regardless of the amount of *saePQRS*::*kan* supernatant included in the incubation (S4 Fig) suggesting the inhibitory factor is not sensitive to *saeRS*-repressed proteinases, and expression is likely transcriptionally regulated by *saeRS*.

## Conclusion

In summary, RNA-Seq showed that the *saeS* ^*P*^ allele of strain Newman affects expression of nearly 125 genes, relative to strains with the *saeS* ^*L*^ allele. The RNA-Seq analyses confirmed that the *saeS* ^*P*^ allele has a profound effect on gene expression, including numerous genes that may play a role in biofilm formation. Genes affected by SaeS^P^ include those that encode autolysins and extracellular nuclease. In accordance, extracellular DNA can be found in culture supernatants of *sae* mutants, but is essentially undetectable in Newman cultures. Inactivation of the Newman *nuc* gene also increased the level of extracellular DNA, but the mutation was insufficient to permit biofilm formation. Expression of the *ica* genes and production of PIA is associated with biofilm formation by some staphylococci but we found no evidence that *sae* affects *ica* expression in strain Newman at least under the conditions we used. Wild type Newman (*saeS* ^*P*^) produces a factor that inhibits biofilm by the producing strain, as well as biofilm formation by other strains of *S*. *aureus*. The inhibitory factor is not produced at a detectable level by a Newman *sae* deleted strain or a Newman mutant expressing SaeS^L^. The inhibitory factor was determined to be a high-molecular weight, thermostable, extracellular protein, but extensive biochemical and genetic analyses have not revealed the identity of the inhibitor. The inhibitory protein appears to be highly potent as we have detected activity in preparations of low protein content. Our results also indicate that the inhibitor is not likely a protease or a group of proteases. We are currently using genetic methods to identify Newman mutants that either do not express or exhibit reduced expression of the inhibitory protein. Without knowing its identity, it is difficult to speculate on the inhibitor’s mechanism of action. However, it is possible that it alters the bacterial surface or inhibits biofilm promoting surface proteins thereby preventing biofilm formation.

## Materials and Methods

### Bacterial strains and culture conditions

The bacterial strains and plasmids used in this study are listed in Table 1. *S*. *aureus* strains were cultivated in Trypticase soy broth without added glucose (TSB-0g) (17 g/L tryptone; 3 g/L soytone; 5 g/L NaCl; 2.5 g/L K_2_HPO_4_ 3H_2_O), TSB (Difco) or tryptic soy agar (TSA) (Difco). Antibiotics were added to *S*. *aureus* culture media, as appropriate, at final concentrations of 10 μg per ml chloramphenicol (Cm), 3 μg per ml tetracycline (Tc), or 50 μg per ml kanamycin (Kn). *Escherichia coli* strain DH5α was used for plasmid construction and maintenance. *E*. *coli* was cultivated in Luria-Bertani broth or agar (Difco) supplemented with, as appropriate, 100 μg per ml penicillin (Pen), 34 μg per ml Cm or 50 μg per ml Kn. The mutants used for screening potential biofilm inhibitor were constructed by transduction from defined *bursa aurealis* transposon mutants in the Nebraska transposon library was obtained from network of Antimicrobial Resistance in *Staphylococcus aureus* (NARSA) program supported under NIAID/NIH. Phage transductions were performed using bacteriophage 52A or bacteriophage 80α to transfer plasmids or transposon insertions.

**Table 1:** Strains and Plasmids used in this study.

Strain	Description	Reference or Source
***S*. *aureus***		
RN4220	Restriction minus strain	J. Iandolo
CYL5876	Newman wild type	T. Foster
CYL11481	Newman *saeS* ^*L*^	[14]
CYL12367	Newman Δ*saePQRS*::*kan*	[24]
CYL11771	Newman Δ*saeRS*	[14]
TB1	Newman deleted for prophage NM3	[43]
TB3	Newman cured of phages NM1, NM2 and NM4	[43]
CYL13095	Newman Δ*saeRS* (pCWSAE50)	This study
CYL13096	Newman Δ*saeRS* (pCWSAE51)	This study
CYL5760	UAMS-1	[50]
CYL11246	USA100	K. Bayles
CYL11247	USA300	K. Bayles
CYL11248	USA500	K. Bayles
NRS678	USA500 NRS678	NARSA
NRS385	USA500 NRS385	NARSA
CYL12147	USA300 FPR3757 Erm^S^	M. Smeltzer
CYL12397	USA300 FPR3757 Erm^S^ Δ*saePQRS*::*kan*	This study
CYL12398	USA300 FPR3757 Erm^S^ Δ*saePQRS*::*kan* (pCWSAE50)	This study
CYL12496	USA300 FPR3757 Erm^S^ Δ*saePQRS*::*kan* (pCWSAE51)	This study
AH2395	Newman Δ*nuc*::*ltrB*	[29]
KB345	RN4220 *lrgAB*::*erm*	[38]
CYL12305	Newman *lrgAB*::*erm*	This study
KB600	RN6390 *spl*::*erm*	[51]
***S*. *epidermidis***		
CYL10028	*S*. *epidermidis* RP12	ATCC
CYL10029	*S*. *epidermidis* RP62A	ATCC
***E*.*coli***		
DH5α	Used for plasmid construction and maintenance	Invitrogen
**Plasmids**		
pCWSAE50	integrative plasmid with *saePQRS* ^*L*^	[24]
pCWSAE51	integrative plasmid with *saePQRS* ^*P*^	[24]

### Biofilm Assay

Biofilm assays were performed as described [11,46] with a few modifications. The medium used was TSB-0g supplemented with 1% NaCl and 0.5% glucose (biofilm medium). Prior to inoculation, microtiter plates were pre-coated with 20% human plasma proteins (Sigma) and incubated for at least one hour at 4°C. After inoculation, plates were incubated at 37°C for 16 h. For experiments that tested anti-biofilm activity of culture supernatants, supernatants were added to fresh biofilm media at 25% vol/vol. Cultures used to harvest supernatants were grown to stationary phase in TSB-0g. To test fractionated culture supernatants for anti-biofilm activity, 20 μl of each fraction was added to each well of a microtiter plate and 180 μl of biofilm medium, pre-inoculated with strain UAMS-1, was added to each well.

### Attachment assays

Stationary phase cultures of strain Newman and the *saePQRS*::*kan* mutant were diluted to an OD_660_ of 0.05, grown to an OD_660_ of 1 and diluted to OD_660_ 0.1 in biofilm medium. The cells were then inoculated into a 24-well plate pre-coated with plasma proteins. Bacteria were allowed to attach for 1 h at 37°C and then were washed, fixed, and stained with crystal violet.

### Protein fractionation

For ammonium sulfate fractionation of culture supernatants, solid ammonium sulfate was added to filter sterilized culture supernatants at concentrations of 30%, 45% or 60% (w/v) and incubated at 4°C for at least one hour. Precipitated proteins were collected by centrifugation at 10,000 x *g* for 30 min. Protein pellets were resuspended in 1X PBS and dialyzed against PBS to remove residual ammonium sulfate.

### Size exclusion and ion exchange chromatography

For chromatography experiments, stationary phase cultures of strain CYL12640 were harvested and concentrated 100-fold using Amicon Ultra centrifugal filters with a 30 kDa molecular weight cut off. For size exclusion chromatography, this sample was separated using a HiPrep 16/60 Sephacryl S-300 HR column (GE Healthcare) without dialysis. For ion exchange chromatography, the sample was separated using a HiTrap Q XL column (GE Healthcare) after dialysis against 50 mM Tris, pH 7.6. For parallel *sae* mutant comparisons, strain CYL12688 was fractionated in the same manner. In-gel trypsin digestion and tandem mass spectrometry for protein identification was performed by the Proteomics core in the Translational Research Institute at the University of Arkansas for Medical Sciences.

### RNA Isolation and rRNA removal

*S*. *aureus* strains were grown overnight in TSB-0g, diluted 1:100 in fresh TSB-0g and grown for 5 h (OD_660_~2.0) in a 10:1 flask:volume ratio. The cultures were mixed with an equal volume of an ice-cold 1:1 mixture of ethanol-acetone and kept frozen at −80°C for at least 2 hours. RNA was isolated as described previously [47]. Isolated RNA was then enriched for mRNA using the MicrobExpress kit (Ambion). mRNA samples were examined for rRNA depletion using a 2100 Agilent Bioanalyzer (Winthrop P. Rockefeller Genomics Core Laboratory at UAMS) or the Oklahoma University Health Science Center, Laboratory for Molecular Biology and Cytometry Research.

### RNA-Seq analysis

Three independently prepared RNA samples from each strain were used for RNA-Seq. Illumina sequencing was performed by the Oklahoma University Health Science Center, Laboratory for Molecular Biology and Cytometry Research, using the Illumina MiSeq sequencer and Illumina TruSeq RNA v2 sample preparation kit and protocols (Illumina Inc). Data analyses were performed using Perkin Elmer’s Genesifter software. Genes exhibiting ≥2-fold changes in expression, which were statistically significant as determined by Student's *t* test (*p* ≤ 0.05), were considered to be differentially expressed under the conditions indicated.

### Real-time RT-PCR

To confirm RNA-Seq data, we selected genes that were regulated by *sae* and assessed their relative expression levels by real-time RT-PCR as described previously [48] using the primer pairs listed in S10 Table. Cycling conditions were as previously described [48].

### Zymographic analysis

For profiling *S*. *aureus* autolysis, cultures were grown in TSB for 3 h (OD660 of ~1.7) and harvested by centrifugation. Surface-associated proteins were prepared with 4% SDS as described [47]. A modified method of Sugai *et al*. [49] was used for bacteriolytic enzyme profiling analysis as previously described [47]. Samples (15 μl) were mixed with 4X SDS sample buffer (final DTT concentration of 12.5 μM), incubated at room temperature for 30 min, and loaded on to 10% SDS-PAGE gels containing heat-killed *S*. *aureus* RN4220 (about 2 mg per ml of gel solution) or heat killed *Micrococcus luteus* (Sigma). After electrophoresis, gels were first washed 3 times each with 250 ml deionized water for 30 min with slow shaking at room temperature and then washed with 250 ml buffer A [50 mM Tris-HCl (pH 7.6), 200 mM NaCl, 5 mM CaCl_2_ at room temperature for 30 min. The gels were incubated overnight with fresh buffer A at 37°C without shaking and then scanned with dark background.

## Supporting Information

S1 Fig A *nuc* or *lrgAB* mutant of strain Newman does not form a biofilm. Stationary phase cultures were diluted to an OD_660_ of 0.05 and inoculated into wells of a microtiter plate. The microtiter plate wells had been precoated with human plasma. After 16 h, biofilms were washed, fixed, and stained with crystal violet.
(TIF)

S2 Fig Strain Newman cured of prophage does not form biofilm. TB3 is a derivative of strain Newman cured of the NM1, NM2 and NM4 prophages (Bae, T. et al. (2006) Prophages of *Staphylococcus aureus* Newman and their contribution to virulence. Mol Microbiol. 62:1035–47). TB1(p*saePQRS* ^*P*^) is deleted for the defective NM3 prophage and then complemented with pCWsae51.
(TIF)

S3 Fig The serine proteinases encoded by the *splABCDEF* operon do not affect biofilm formation. (A) Biofilm formation by wild type Newman, Newman *ΔsaeRS* and a Newman derivative deleted for the *splABDCEF* operon (*spl*::*erm*) were diluted to an OD_660_ of 0.05 and inoculated into wells of a microtiter plate. The microtiter plate wells had been precoated with human plasma. After 16 h, biofilms were washed, fixed, and stained with crystal violet. (B) Inhibition of biofilm formation; stationary phase culture supernatants of strain Newman and its derivatives were harvested, filter sterilized and added to microtiter plate wells preinoculated with *S*. *aureus* UAMS-1 suspended in biofilm medium. The source of each culture supernatant is listed to the right of the picture.
(TIF)

S4 Fig The biofilm inhibitory protein is not degraded by a proteinase in the *saePQRS*::*kan* mutant. Stationary phase culture supernatants of Newman and Newman *saePQRS*::*kan* were combined at the indicated ratios and incubated overnight at 37°C. Proteins were TCA precipitated, dialyzed and added to biofilm media. Anti-biofilm activity was tested against strain UAMS-1. The *saePQRS*::*kan* supernatant did not inactivate the Newman supernatant.
(TIF)

S1 Table Proteins identified and tested for biofilm inhibition. (DOC)

S2 Table Genes down regulated in CYL11771 (Δ*saeRS*) relative to wild-type Newman (CYL5876). (DOCX)

S3 Table Genes down regulated in CYL11771 (Δ*saeRS*) relative to CYL11481 (*saeS* ^*L*^). (DOCX)

S4 Table Genes down regulated in CYL11481 (*saeS* ^*L*^) relative to wild-type Newman (CYL5876). (DOCX)

S5 Table Genes up regulated in CYL11771 (Δ*saeRS*) relative to wild-type Newman (CYL5876). (DOCX)

S6 Table Genes up regulated in CYL11771 (Δ*saeRS*) relative to CYL11481 (*saeS* ^*L*^). (DOCX)

S7 Table Genes up regulated in CYL11481 (*saeS* ^*L*^) relative to wild-type Newman (CYL5876). (DOCX)

S8 Table Newman genes exhibiting an apparent dose-dependent response to SaeRS levels. (DOCX)

S9 Table Bacteriophage genes up regulated by *saeS* ^*P*^. (DOCX)

S10 Table Oligonucleotide primers used for real-time PCR. (DOCX)
