# Supplementary figures and images for: SaeRS-Dependent Inhibition of Biofilm Formation in Staphylococcus aureus Newman
Source: PLoS One. 2015 Apr 8;10(4):e0123027. doi: 10.1371/journal.pone.0123027 (PMC4390220; doi:10.1371/journal.pone.0123027)

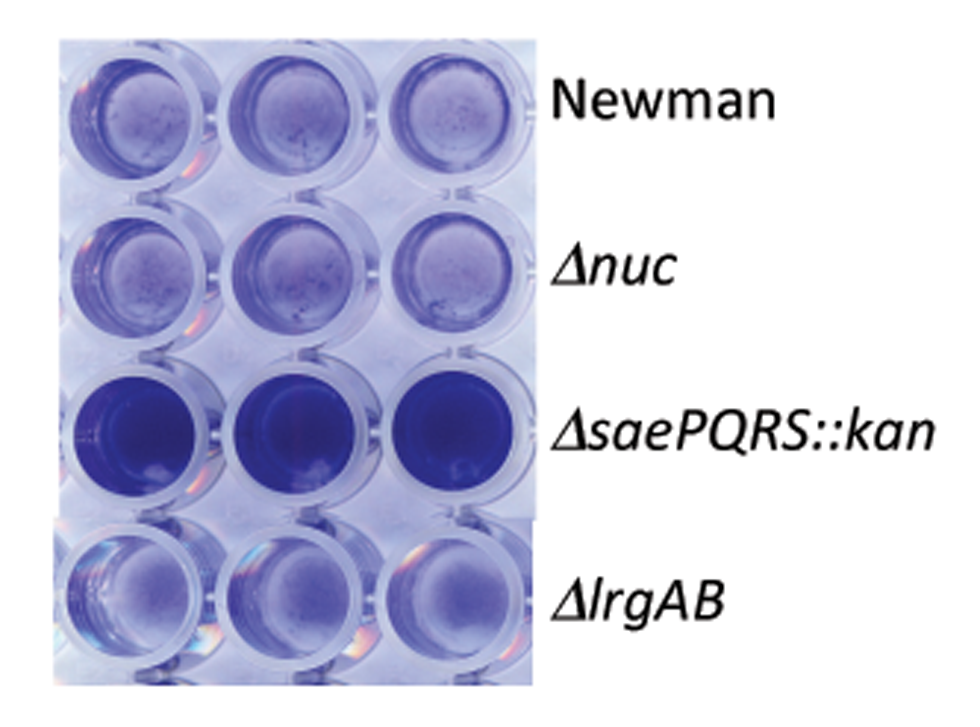

Supplement: S1 Fig — Stationary phase cultures were diluted to an OD660 of 0.05 and inoculated into wells of a microtiter plate. The microtiter plate wells had been precoated with human plasma. After 16 h, biofilms were washed, fixed, and stained with crystal violet. (TIF) [file pone.0123027.s001.tif]

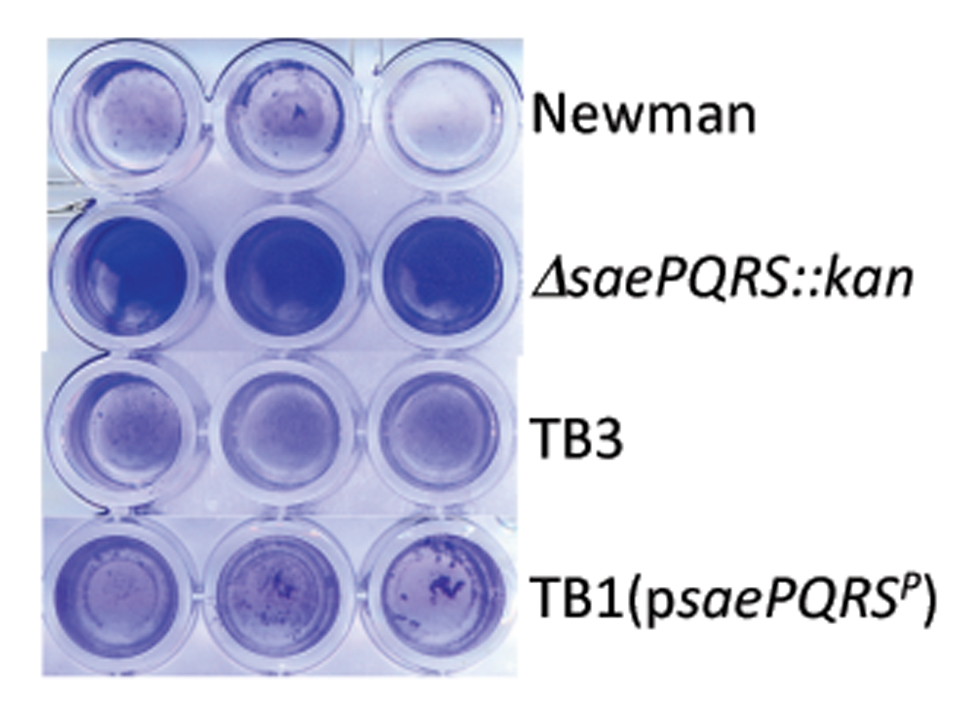

Supplement: S2 Fig — TB3 is a derivative of strain Newman cured of the NM1, NM2 and NM4 prophages (Bae, T. et al. (2006) Prophages of Staphylococcus aureus Newman and their contribution to virulence. Mol Microbiol. 62:1035–47). TB1(psaePQRS P) is deleted for the defective NM3 prophage and then complemented with pCWsae51. (TIF) [file pone.0123027.s002.tif]

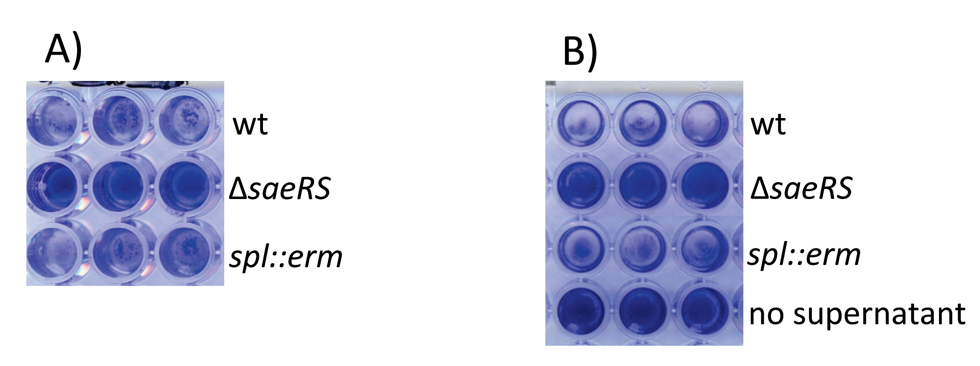

Supplement: S3 Fig — (A) Biofilm formation by wild type Newman, Newman ΔsaeRS and a Newman derivative deleted for the splABDCEF operon (spl::erm) were diluted to an OD660 of 0.05 and inoculated into wells of a microtiter plate. The microtiter plate wells had been precoated with human plasma. After 16 h, biofilms were washed, fixed, and stained with crystal violet. (B) Inhibition of biofilm formation; stationary phase culture supernatants of strain Newman and its derivatives were harvested, filter sterilized and added to microtiter plate wells preinoculated with S. aureus UAMS-1 suspended in biofilm medium. The source of each culture supernatant is listed to the right of the picture. (TIF) [file pone.0123027.s003.tif]

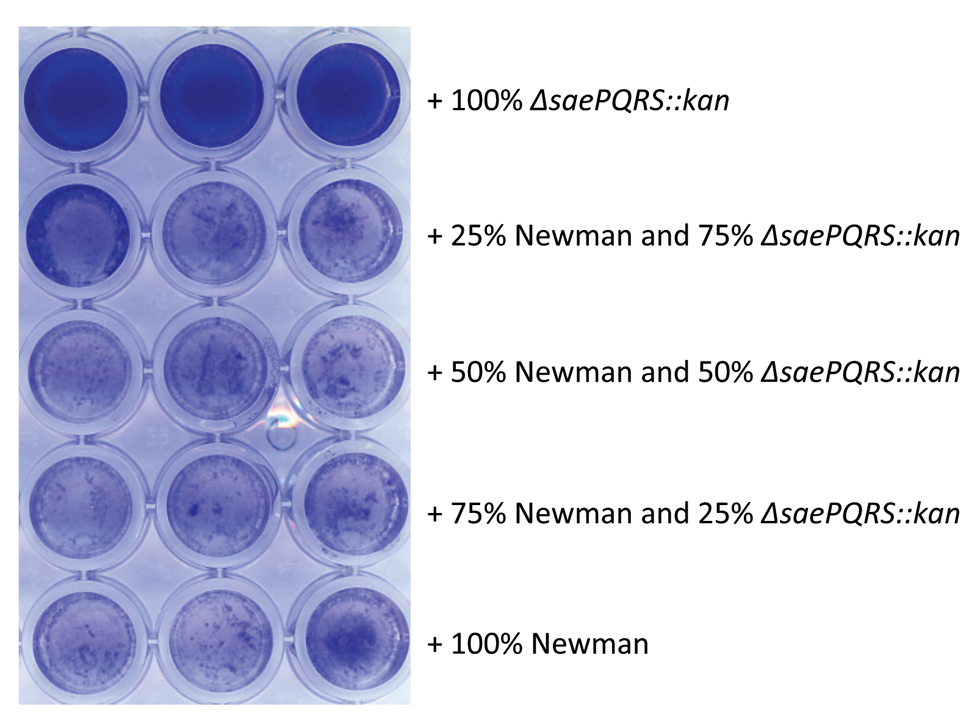

Supplement: S4 Fig — Stationary phase culture supernatants of Newman and Newman saePQRS::kan were combined at the indicated ratios and incubated overnight at 37°C. Proteins were TCA precipitated, dialyzed and added to biofilm media. Anti-biofilm activity was tested against strain UAMS-1. The saePQRS::kan supernatant did not inactivate the Newman supernatant. (TIF) [file pone.0123027.s004.tif]
