# Supplementary material for: SaeRS-Dependent Inhibition of Biofilm Formation in Staphylococcus aureus Newman
Source: PLoS One. 2015 Apr 8;10(4):e0123027. doi: 10.1371/journal.pone.0123027 (PMC4390220; doi:10.1371/journal.pone.0123027)
Supplement: S1 Table — (DOC) [file pone.0123027.s005.doc]

| **Table S1. Potential biofilm inhibitory proteins identified using chromatography that were eliminated based on genetic tests.** | |
| --- | --- |
| **Locus tag/ gene name** | **Annotation** |
| NWMN_0055 (*spa*) | surface protein A |
| NWMN_0087 | hypothetical protein |
| NWMN_0166 (*coa*) | coagulase precursor |
| NWMN_0178 | hypothetical protein |
| NWMN_0249 | 5'-nucleotidase, lipoprotein e(P4) family protein |
| NWMN_0262 (*geh*) | truncated triacylglycerol lipase |
| NWMN_0293 | hypothetical protein |
| NWMN_0401 | hypothetical protein |
| NWMN_0533 | hypothetical protein |
| NWMN_0601 | hypothetical protein |
| NWMN_0677 (*saeP*) | hypothetical protein |
| NWMN_0685 | hypothetical protein |
| NWMN_0757 | hypothetical protein |
| NWMN_0760 (*nuc*) | thermonuclease precursor |
| NWMN_0824 | hypothetical protein |
| NWMN_0892 (*htrA*) | serine protease |
| NWMN_0922 (*atl*) | autolysin |
| NWMN_0961 (*pdhC*) | branched-chain alpha-keto acid dehydrogenase subunit E2 |
| NWMN_1073 (*hla*) | alpha-hemolysin |
| NWMN_1278 | hypothetical protein |
| NWMN_1345 (*ebh*) | cell surface protein |
| NWMN_1621 | hypothetical protein |
| NWMN_1718 (*lukD*) | leukocidin |
| NWMN_1846 (*sspB*) | staphopain thiol proteinase |
| NWMN_1872 (*map*/*eap*) | MHC class II analog protein |
| NWMN_1926 (*hlb*) | truncated beta-hemolysin |
| NWMN_1928 | hypothetical protein |
| NWMN_1940 (*sdrH*) | SdrH protein |
| NWMN_2109 | truncated MHC II analog protein |
| NWMN_2199 (*ssaA*) | secretory antigen precursor |
| NWMN_2270 | hypothetical protein |
| NWMN_2317 (*sbi*) | IgG-binding protein Sbi |
| NWMN_2319 (*hlgC*) | gamma-hemolysin component C |
| NWMN_2320 (*hlgB*) | gamma-hemolysin component B |
| NWMN_2392 | hypothetical protein |
| NWMN_2397 (*fnbB*) | fibronectin-binding protein |
| NWMN_2399 (*fnbA*) | fibronectin-binding protein |
| NWMN_2529 (*clfB*) | clumping factor B |
| NWMN_2536 (*aur*) | aureolysin (metalloproteinase) |
| NWMN_2543 | hypothetical protein |
| NWMN_2569 (*lip*) | triacylglycerol lipase |
