# Supplementary material for: SaeRS-Dependent Inhibition of Biofilm Formation in Staphylococcus aureus Newman
Source: PLoS One. 2015 Apr 8;10(4):e0123027. doi: 10.1371/journal.pone.0123027 (PMC4390220; doi:10.1371/journal.pone.0123027)
Supplement: S2 Table — (DOCX) [file pone.0123027.s006.docx]

**Table S2. Genes down regulated in CYL11771 (*ΔsaeRS*) relative to wild-type Newman (CYL5876).**

| **Fold-change** | **Gene Name** | **NCBI ID** | **Locus tag** |
| --- | --- | --- | --- |
| 2.04 | hypothetical protein | 5332473 | NWMN_0009 |
| 2.04 | hypothetical protein | 5329921 | NWMN_0010 |
| 2.55 | hypothetical protein | 5329931 | NWMN_0026 |
| 2.13 | hypothetical protein | 5329936 | NWMN_0031 |
| 2.09 | hypothetical protein | 5329937 | NWMN_0032 |
| 2.09 | hypothetical protein | 5329939 | NWMN_0034 |
| 2.22 | hypothetical protein | 5329941 | NWMN_0036 |
| 11.42 | immunoglobulin G binding protein A precursor (protein A) | 5332074 | NWMN_0055 |
| 2.45 | accessory regulator A-like protein | 5332493 | NWMN_0056 |
| 2.88 | acetoin reductase | 5329961 | NWMN_0071 |
| 4.42 | hypothetical protein | 5332492 | NWMN_0078 |
| 3.66 | ABC transport ATP-binding protein | 5329973 | NWMN_0086 |
| 2.37 | hypothetical protein | 5329974 | NWMN_0087 |
| 3.93 | hypothetical protein | 5329982 | NWMN_0112 |
| 2.7 | hypothetical protein | 5329984 | NWMN_0115 |
| 2.07 | formate dehydrogenase | 5329990 | NWMN_0121 |
| 15.86 | hypothetical protein | 5330025 | NWMN_0165 |
| 14.61 | staphylocoagulase precursor | 5330026 | NWMN_0166 |
| 4 | autolysin sensor histidine kinase (LytS) | 5332453 | NWMN_0194 |
| 61.49 | murein hydrolase regulator LrgA | 5332113 | NWMN_0196 |
| 61.49 | antiholin-like protein LrgB | 5332099 | NWMN_0197 |
| 2.36 | hypothetical protein | 5330081 | NWMN_0238 |
| 2.32 | hypothetical protein | 5330082 | NWMN_0239 |
| 2.13 | branched-chain amino acid transport system II carrier protein | 5330091 | NWMN_0248 |
| 2.28 | hypothetical protein | 5330099 | NWMN_0256 |
| 2.16 | hypothetical protein | 5332194 | NWMN_0258 |
| 2.12 | hypothetical protein | 5330101 | NWMN_0259 |
| 2.5 | N-acetylmannosamine-6-phosphate 2-epimerase | 5330102 | NWMN_0260 |
| 2.22 | hypothetical protein | 5332195 | NWMN_0261 |
| 18.25 | truncated triacylglycerol lipase precursor | 5330103 | NWMN_0262 |
| 6.08 | integrase | 5331946 | NWMN_0263 |
| 5.26 | hypothetical protein | 5330167 | NWMN_0328 |
| 2.16 | glyoxalase family protein | 5330169 | NWMN_0330 |
| 2.14 | luciferase-like monooxygenase | 5330170 | NWMN_0331 |
| 4.4 | hypothetical protein | 5330175 | NWMN_0336 |
| 4.4 | hypothetical protein | 5330176 | NWMN_0337 |
| 4.4 | hypothetical protein | 5330177 | NWMN_0338 |
| 2.73 | hypothetical protein | 5330185 | NWMN_0347 |
| 6.7 | 5-methyl-tetrahydropteroyltriglutamate--homocysteine S-methyltransferase | 5330186 | NWMN_0348 |
| 6.7 | bifunctional homocysteine S-methyltransferase/5,10-methylenetetrahydrofolate reductase | 5332126 | NWMN_0349 |
| 6.7 | trans-sulfuration enzyme family protein | 5332127 | NWMN_0350 |
| 6.7 | Cys/Met metabolism PLP-dependent enzyme | 5330187 | NWMN_0351 |
| 7.78 | hypothetical protein | 5330188 | NWMN_0352 |
| 2.64 | hypothetical protein | 5330195 | NWMN_0361 |
| 28.22 | hypothetical protein | 5330196 | NWMN_0362 |
| 2.28 | sodium:dicarboxylate symporter family protein | 5330206 | NWMN_0374 |
| 3.68 | hypothetical protein | 5330208 | NWMN_0376 |
| 5.21 | hypothetical protein | 5332548 | NWMN_0385 |
| 4.98 | superantigen-like protein | 5332476 | NWMN_0389 |
| 452.36 | hypothetical protein | 5330217 | NWMN_0402 |
| 2.37 | cobalamin synthesis protein | 5330223 | NWMN_0417 |
| 2.17 | NADH dehydrogenase subunit 5 | 5331831 | NWMN_0418 |
| 3.45 | ABC transporter permease | 5330229 | NWMN_0427 |
| 3.07 | glutamate synthase, large subunit | 5331963 | NWMN_0436 |
| 2.18 | glutamate synthase subunit beta | 5331962 | NWMN_0437 |
| 2.02 | PTS system, trehalose-specific IIBC component | 5331964 | NWMN_0438 |
| 2.26 | hypothetical protein | 5330240 | NWMN_0447 |
| 2.37 | hypothetical protein | 5332065 | NWMN_0457 |
| 2.38 | 4-diphosphocytidyl-2-C-methyl-D-erythritol kinase | 5332592 | NWMN_0458 |
| 2.05 | Ser-Asp rich fibrinogen/bone sialoprotein-binding protein SdrC | 5330275 | NWMN_0523 |
| 2.14 | hypothetical protein | 5330280 | NWMN_0531 |
| 2.21 | hypothetical protein | 5330284 | NWMN_0535 |
| 3.95 | hypothetical protein | 5330288 | NWMN_0542 |
| 3.97 | hypothetical protein | 5330323 | NWMN_0585 |
| 5.47 | hypothetical protein | 5330324 | NWMN_0586 |
| 2.01 | iron dependent repressor | 5330334 | NWMN_0604 |
| 2.41 | hypothetical protein | 5330360 | NWMN_0644 |
| 3.65 | hypothetical protein | 5330367 | NWMN_0651 |
| 551.34 | sensor histidine kinase SaeS | 5330385 | NWMN_0674 |
| 551.34 | DNA-binding response regulator SaeR | 5332432 | NWMN_0675 |
| 551.34 | hypothetical protein | 5332431 | NWMN_0676 |
| 864.3 | hypothetical protein | 5330386 | NWMN_0677 |
| 2.51 | osmoprotectant ABC transporter ATP-binding protein | 5332344 | NWMN_0690 |
| 2.51 | ABC transporter permease | 5330396 | NWMN_0691 |
| 5.07 | hypothetical protein | 5330428 | NWMN_0738 |
| 25.31 | extracellular matrix and plasma binding protein | 5330439 | NWMN_0758 |
| 37.1 | thermonuclease precursor | 5330440 | NWMN_0760 |
| 8.51 | cold-shock protein CSD family protein | 5330441 | NWMN_0761 |
| 2.05 | hypothetical protein | 5330443 | NWMN_0764 |
| 3.5 | hypothetical protein | 5330444 | NWMN_0765 |
| 2.81 | hypothetical protein | 5330448 | NWMN_0769 |
| 3.75 | hypothetical protein | 5330463 | NWMN_0784 |
| 2.2 | FeS assembly ATPase SufC | 5330464 | NWMN_0785 |
| 3.17 | oligopeptide ABC transporter ATP-binding protein | 5331738 | NWMN_0863 |
| 3.17 | hypothetical protein | 5330508 | NWMN_0864 |
| 3.17 | transcriptional regulator Spx | 5332568 | NWMN_0867 |
| 3.5 | hypothetical protein | 5330540 | NWMN_0901 |
| 2.35 | aminotransferase, class I | 5332508 | NWMN_0919 |
| 4.98 | AtlR autolysin transcriptional regulator | 5330555 | NWMN_0921 |
| 2.49 | acetyltransferase, GNAT family protein | 5331762 | NWMN_0923 |
| 2.3 | hypothetical protein | 5330558 | NWMN_0925 |
| 2.06 | phosphocarrier protein HPr | 5330567 | NWMN_0949 |
| 2.08 | hypothetical protein | 5330606 | NWMN_1002 |
| 2.61 | glutamate racemase | 5332459 | NWMN_1063 |
| 2.46 | nucleoside-triphosphatase | 5330651 | NWMN_1064 |
| 2.46 | hypothetical protein | 5330652 | NWMN_1065 |
| 978.01 | hypothetical protein | 5330653 | NWMN_1066 |
| 20.36 | formyl peptide receptor-like 1 inhibitory protein | 5330654 | NWMN_1067 |
| 3.42 | hypothetical protein | 5330655 | NWMN_1068 |
| 852.35 | hypothetical protein | 5330656 | NWMN_1069 |
| 409.52 | hypothetical protein | 5330657 | NWMN_1070 |
| 551.1 | alpha-hemolysin precursor | 5330660 | NWMN_1073 |
| 2.15 | hypothetical protein | 5332007 | NWMN_1074 |
| 6.26 | superantigen-like protein | 5330661 | NWMN_1075 |
| 4.36 | superantigen-like protein | 5330662 | NWMN_1076 |
| 7.37 | superantigen-like protein | 5330663 | NWMN_1077 |
| 2.27 | anti protein | 5331661 | NWMN_1084 |
| 2.05 | cell division protein MraZ | 5330671 | NWMN_1088 |
| 2.22 | S-adenosyl-methyltransferase MraW | 5330672 | NWMN_1089 |
| 2.21 | hypothetical protein | 5330673 | NWMN_1098 |
| 2.21 | hypothetical protein | 5330674 | NWMN_1099 |
| 2.4 | guanylate kinase | 5330685 | NWMN_1120 |
| 2.94 | hypothetical protein | 5330727 | NWMN_1225 |
| 4.95 | hypothetical protein | 5330728 | NWMN_1226 |
| 2.28 | hypothetical protein | 5330731 | NWMN_1229 |
| 3.34 | aspartate kinase | 5330740 | NWMN_1239 |
| 2.53 | homoserine dehydrogenase | 5332539 | NWMN_1240 |
| 2.71 | threonine synthase | 5332129 | NWMN_1241 |
| 2.9 | LexA repressor | 5330745 | NWMN_1251 |
| 2.58 | indole-3-glycerol-phosphate synthase | 5332564 | NWMN_1282 |
| 2.58 | N-(5-phosphoribosyl)anthranilate isomerase | 5332563 | NWMN_1283 |
| 2.58 | tryptophan synthase subunit beta | 5332566 | NWMN_1284 |
| 2.58 | tryptophan synthase subunit alpha | 5332562 | NWMN_1285 |
| 3.28 | phosphate ABC transporter phosphate-binding protein PstS | 5330771 | NWMN_1300 |
| 2.59 | aspartate kinase | 5330774 | NWMN_1304 |
| 3.2 | dihydrodipicolinate synthase | 5331759 | NWMN_1306 |
| 3.2 | dihydrodipicolinate reductase | 5331853 | NWMN_1307 |
| 3.37 | tetrahydrodipicolinate acetyltransferase (dapD) | 5331854 | NWMN_1308 |
| 4.09 | two-component sensor histidine kinase | 5332514 | NWMN_1327 |
| 4.09 | arlR, two-component response regulator | 5330787 | NWMN_1328 |
| 2.21 | hypothetical protein (ebh) | 5331883 | NWMN_1345 |
| 41.35 | amino acid permease | 5330798 | NWMN_1347 |
| 92.22 | threonine dehydratase | 5330799 | NWMN_1348 |
| 46.51 | alanine dehydrogenase | 5332036 | NWMN_1349 |
| 2.54 | hypothetical protein | 5330852 | NWMN_1436 |
| 2.08 | hypothetical protein | 5330856 | NWMN_1442 |
| 2.87 | DNA internalization-related competence protein ComEC/Rec2 | 5332011 | NWMN_1490 |
| 2.98 | hypothetical protein | 5330917 | NWMN_1531 |
| 2.04 | glutamyl-tRNA reductase | 5331994 | NWMN_1566 |
| 4.59 | hypothetical protein | 5330987 | NWMN_1670 |
| 2.96 | hypothetical protein | 5330990 | NWMN_1673 |
| 2.93 | hypothetical protein | 5331000 | NWMN_1688 |
| 2.72 | hypothetical protein | 5331001 | NWMN_1689 |
| 2.41 | hypothetical protein | 5331008 | NWMN_1696 |
| 8.2 | serine protease SplF | 5332017 | NWMN_1701 |
| 5.31 | serine protease SplE | 5332499 | NWMN_1702 |
| 8.23 | serine protease SplD | 5332498 | NWMN_1703 |
| 9.93 | serine protease SplC | 5332497 | NWMN_1704 |
| 16.41 | serine protease SplB | 5332496 | NWMN_1705 |
| 2.3 | hypothetical protein | 5331022 | NWMN_1732 |
| 2.58 | staphopain thiol proteinase | 5332301 | NWMN_1847 |
| 2.38 | hypothetical protein | 5332510 | NWMN_1848 |
| 3.79 | hypothetical protein | 5331129 | NWMN_1860 |
| 2.66 | hypothetical protein pmtA | 5331135 | NWMN_1866 |
| 2.66 | ABC transporter ATP-binding protein | 5331136 | NWMN_1867 |
| 2.66 | hypothetical protein | 5331137 | NWMN_1868 |
| 242.9 | MHC class II analog protein (map) | 5331141 | NWMN_1872 |
| 9.88 | hypothetical protein | 5331142 | NWMN_1874 |
| 9.88 | hypothetical protein | 5331143 | NWMN_1875 |
| 98 | complement inhibitor (scn) | 5331144 | NWMN_1876 |
| 145.03 | chemotaxis-inhibiting protein (chp) | 5332454 | NWMN_1877 |
| 4.25 | hypothetical protein | 5331155 | NWMN_1891 |
| 4.25 | hypothetical protein | 5331156 | NWMN_1892 |
| 4.25 | phage head-tail adaptor | 5331157 | NWMN_1893 |
| 4.25 | hypothetical protein | 5331158 | NWMN_1894 |
| 4.25 | hypothetical protein | 5331159 | NWMN_1895 |
| 4.26 | phage major capsid protein | 5331160 | NWMN_1896 |
| 2.4 | phage Clp-like protease | 5331161 | NWMN_1897 |
| 2.4 | phage portal protein | 5331162 | NWMN_1898 |
| 2.91 | hypothetical protein | 5331177 | NWMN_1913 |
| 2.91 | hypothetical protein | 5331178 | NWMN_1914 |
| 2.91 | hypothetical protein | 5331179 | NWMN_1915 |
| 41.13 | leukocidin/hemolysin toxin subunit F | 5331190 | NWMN_1927 |
| 64.12 | leukocidin/hemolysin toxin subunit S | 5332104 | NWMN_1928 |
| 2.67 | hypothetical protein | 5331193 | NWMN_1932 |
| 3.52 | hypothetical protein | 5332465 | NWMN_1941 |
| 2.02 | staphylococcal accessory gene regulator A | 5331721 | NWMN_1946 |
| 2.85 | isopropylmalate isomerase small subunit | 5332079 | NWMN_1966 |
| 2.52 | potassium-transporting ATPase subunit C | 5331217 | NWMN_1980 |
| 2.54 | potassium-transporting ATPase subunit B | 5332062 | NWMN_1981 |
| 3.53 | hypothetical protein | 5332175 | NWMN_2005 |
| 2.72 | hypothetical protein | 5331265 | NWMN_2073 |
| 3.15 | putative transposase for IS1272 | 5331275 | NWMN_2083 |
| 3.15 | putative transposase for IS1272 | 5331276 | NWMN_2084 |
| 2.63 | PTS system, lactose-specific IIBC component | 5332072 | NWMN_2094 |
| 2.83 | tagatose 1,6-diphosphate aldolase | 5332071 | NWMN_2096 |
| 2.03 | hypothetical protein | 5332569 | NWMN_2122 |
| 2.03 | cobalt transporter ATP-binding subunit | 5331301 | NWMN_2123 |
| 2.03 | cobalt transporter ATP-binding subunit | 5331302 | NWMN_2124 |
| 2.18 | hypothetical protein | 5331343 | NWMN_2219 |
| 2.69 | hypothetical protein | 5331352 | NWMN_2228 |
| 2.32 | peptidase M20/M25/M40 family protein | 5331354 | NWMN_2230 |
| 2.04 | teicoplanin resistance operon transcriptional regulator TcaR | 5332529 | NWMN_2258 |
| 2.46 | hypothetical protein | 5331399 | NWMN_2285 |
| 2.35 | MerR family regulatory protein | 5331404 | NWMN_2290 |
| 2.28 | cation efflux family protein | 5331973 | NWMN_2316 |
| 102.19 | immunoglobulin G-binding protein Sbi | 5331420 | NWMN_2317 |
| 127.03 | gamma-hemolysin component A | 5332443 | NWMN_2318 |
| 226.61 | gamma-hemolysin component C | 5332008 | NWMN_2319 |
| 65.81 | gamma hemolysin, component B | 5332010 | NWMN_2320 |
| 5.78 | hypothetical protein | 5332009 | NWMN_2321 |
| 2.78 | Na+/H+ antiporter family protein | 5331431 | NWMN_2338 |
| 2.49 | oligopeptide permease, peptide-binding protein | 5332219 | NWMN_2364 |
| 2.54 | hypothetical protein | 5331449 | NWMN_2367 |
| 6.29 | hypothetical protein | 5331459 | NWMN_2377 |
| 2.33 | hypothetical protein | 5331474 | NWMN_2392 |
| 8.03 | C-terminal part of fibronectin binding protein B | 5331979 | NWMN_2396 |
| 6 | fibronectin binding protein B precursor | 5331476 | NWMN_2397 |
| 6.11 | fibronectin binding protein A precursor | 5331477 | NWMN_2399 |
| 2.82 | hypothetical protein | 5331482 | NWMN_2408 |
| 15.81 | hypothetical protein | 5331487 | NWMN_2413 |
| 2.41 | hypothetical protein | 5331490 | NWMN_2417 |
| 2.92 | sortase | 5331499 | NWMN_2426 |
| 2.59 | hypothetical protein | 5331500 | NWMN_2428 |
| 3.31 | hypothetical protein | 5331502 | NWMN_2432 |
| 2.15 | metallo-beta-lactamase superfamily protein | 5331563 | NWMN_2512 |
| 15.5 | clumping factor B precursor | 5331889 | NWMN_2529 |
| 2.04 | mannose-6-phosphate isomerase | 5331581 | NWMN_2541 |
| 2.66 | lipase precursor | 5332031 | NWMN_2569 |
| 3.1 | hypothetical protein | 5331608 | NWMN_2584 |
| 3.93 | 5S ribosomal RNA | 5331635 | NWMN_rRNA07 |
| 3.77 | tRNA | 5331663 | NWMN_tRNA18 |
| 3.75 | tRNA | 5331665 | NWMN_tRNA20 |
| 3.54 | tRNA | 5331666 | NWMN_tRNA21 |
