# Supplementary material for: SaeRS-Dependent Inhibition of Biofilm Formation in Staphylococcus aureus Newman
Source: PLoS One. 2015 Apr 8;10(4):e0123027. doi: 10.1371/journal.pone.0123027 (PMC4390220; doi:10.1371/journal.pone.0123027)
Supplement: S3 Table — (DOCX) [file pone.0123027.s007.docx]

**Table S3. Genes down regulated in CYL11771 ( ΔsaeRS) relative to CYL11481 (saeS^L^).**

| **Fold-change** | **Gene Name** | **NCBI ID** | **Locus tag** |
| --- | --- | --- | --- |
| 2.59 | hypothetical protein | 5329936 | NWMN_0031 |
| 2.7 | hypothetical protein | 5330018 | NWMN_0157 |
| 3.12 | hypothetical protein | 5330025 | NWMN_0165 |
| 2.74 | murein hydrolase regulator LrgA | 5332113 | NWMN_0196 |
| 2.74 | antiholin-like protein LrgB | 5332099 | NWMN_0197 |
| 2.39 | hypothetical protein | 5330051 | NWMN_0207 |
| 5.3 | truncated triacylglycerol lipase precursor | 5330103 | NWMN_0262 |
| 4.75 | hypothetical protein | 5330188 | NWMN_0352 |
| 2.08 | hypothetical protein | 5330196 | NWMN_0362 |
| 2.32 | hypothetical protein | 5332548 | NWMN_0385 |
| 14.89 | hypothetical protein | 5330217 | NWMN_0402 |
| 2.37 | mevalonate kinase | 5330296 | NWMN_0553 |
| 27.37 | sensor histidine kinase SaeS | 5330385 | NWMN_0674 |
| 27.37 | DNA-binding response regulator SaeR | 5332432 | NWMN_0675 |
| 27.37 | hypothetical protein | 5332431 | NWMN_0676 |
| 23.91 | hypothetical protein | 5330386 | NWMN_0677 |
| 4.49 | cold-shock protein CSD family protein | 5330441 | NWMN_0761 |
| 2.56 | oligopeptide ABC transporter ATP-binding protein | 5331738 | NWMN_0863 |
| 2.56 | hypothetical protein | 5330508 | NWMN_0864 |
| 3.24 | hypothetical protein | 5330540 | NWMN_0901 |
| 2.46 | cysteine protease precursor | 5332121 | NWMN_0916 |
| 58.25 | hypothetical protein | 5330653 | NWMN_1066 |
| 2.18 | hypothetical protein | 5330655 | NWMN_1068 |
| 34.41 | hypothetical protein | 5330656 | NWMN_1069 |
| 14.85 | hypothetical protein | 5330657 | NWMN_1070 |
| 854.5 | alpha-hemolysin precursor | 5330660 | NWMN_1073 |
| 5.12 | superantigen-like protein | 5330661 | NWMN_1075 |
| 2.06 | S-adenosyl-methyltransferase MraW | 5330672 | NWMN_1089 |
| 2.23 | hypothetical protein | 5330686 | NWMN_1124 |
| 2.88 | glutamine synthetase repressor | 5330720 | NWMN_1216 |
| 4.29 | hypothetical protein | 5330728 | NWMN_1226 |
| 2.08 | hypothetical protein | 5330737 | NWMN_1235 |
| 3.87 | phosphate ABC transporter permease | 5330770 | NWMN_1299 |
| 4.08 | phosphate ABC transporter phosphate-binding protein PstS | 5330771 | NWMN_1300 |
| 2.22 | two-component sensor histidine kinase | 5332514 | NWMN_1327 |
| 2.22 | two-component response regulator | 5330787 | NWMN_1328 |
| 2.08 | hypothetical protein (ebh) | 5331883 | NWMN_1345 |
| 2.46 | hypothetical protein | 5330873 | NWMN_1463 |
| 3.2 | DNA internalization-related competence protein ComEC/Rec2 | 5332011 | NWMN_1490 |
| 2.69 | hypothetical protein | 5330917 | NWMN_1531 |
| 2.64 | hypothetical protein | 5330987 | NWMN_1670 |
| 8.03 | serine protease SplF | 5332017 | NWMN_1701 |
| 5.25 | serine protease SplE | 5332499 | NWMN_1702 |
| 8.3 | serine protease SplD | 5332498 | NWMN_1703 |
| 5.67 | serine protease SplC | 5332497 | NWMN_1704 |
| 10.53 | serine protease SplB | 5332496 | NWMN_1705 |
| 2 | hypothetical protein | 5331022 | NWMN_1732 |
| 2.06 | hypothetical protein | 5331055 | NWMN_1768 |
| 4.52 | MHC class II analog protein | 5331141 | NWMN_1872 |
| 4.04 | complement inhibitor SCIN | 5331144 | NWMN_1876 |
| 5.38 | chemotaxis-inhibiting protein CHIPS | 5332454 | NWMN_1877 |
| 2.05 | hypothetical protein | 5331155 | NWMN_1891 |
| 2.05 | hypothetical protein | 5331156 | NWMN_1892 |
| 2.05 | phage head-tail adaptor | 5331157 | NWMN_1893 |
| 2.05 | hypothetical protein | 5331158 | NWMN_1894 |
| 2.05 | hypothetical protein | 5331159 | NWMN_1895 |
| 2.97 | phage dUTP pyrophosphatase | 5331168 | NWMN_1904 |
| 12.17 | leukocidin/hemolysin toxin subunit F | 5331190 | NWMN_1927 |
| 11.82 | leukocidin/hemolysin toxin subunit S | 5332104 | NWMN_1928 |
| 4.27 | ammonium transporter | 5332456 | NWMN_1950 |
| 4.61 | dihydroxy-acid dehydratase | 5331211 | NWMN_1960 |
| 3.79 | potassium-transporting ATPase subunit A | 5332061 | NWMN_1982 |
| 3.2 | hypothetical protein | 5332175 | NWMN_2005 |
| 2.13 | thymidine kinase | 5332287 | NWMN_2023 |
| 3.51 | PTS system, lactose-specific IIBC component | 5332072 | NWMN_2094 |
| 8.79 | immunoglobulin G-binding protein Sbi | 5331420 | NWMN_2317 |
| 2.04 | gamma-hemolysin component A | 5332443 | NWMN_2318 |
| 11.24 | gamma-hemolysin component C | 5332008 | NWMN_2319 |
| 4.47 | gamma hemolysin, component B | 5332010 | NWMN_2320 |
| 3.65 | hypothetical protein | 5331487 | NWMN_2413 |
| 4.01 | secretory antigen precursor SsaA-like protein | 5331525 | NWMN_2466 |
| 3.8 | hypothetical protein | 5331553 | NWMN_2502 |
| 3 | hypothetical protein | 5331608 | NWMN_2584 |
| 2.41 | 5S ribosomal RNA | 5331635 | NWMN_rRNA07 |
| 2.17 | tRNA | 5331649 | NWMN_tRNA04 |
| 2.11 | tRNA | 5331650 | NWMN_tRNA05 |
| 2.22 | tRNA | 5331652 | NWMN_tRNA07 |
| 3.03 | tRNA | 5331666 | NWMN_tRNA21 |
