# Supplementary material for: SaeRS-Dependent Inhibition of Biofilm Formation in Staphylococcus aureus Newman
Source: PLoS One. 2015 Apr 8;10(4):e0123027. doi: 10.1371/journal.pone.0123027 (PMC4390220; doi:10.1371/journal.pone.0123027)
Supplement: S4 Table — (DOCX) [file pone.0123027.s008.docx]

**Table S4. Genes down regulated in CYL11481 (saeS^L^) relative to wild type Newman (CYL5876).**

| **Fold change** | **Gene Name** | **NCBI ID** | **locus tag** |
| --- | --- | --- | --- |
| 2.03 | acetoin reductase | 5329961 | NWMN_0071 |
| 2.51 | hypothetical protein | 5329982 | NWMN_0112 |
| 2.21 | oligopeptide ABC transporter permease | 5330006 | NWMN_0144 |
| 2.14 | RGD-containing lipoprotein | 5330008 | NWMN_0146 |
| 7.4 | staphylocoagulase precursor | 5330026 | NWMN_0166 |
| 3.26 | autolysin sensor histidine kinase | 5332453 | NWMN_0194 |
| 2.27 | two-component response regulator | 5332114 | NWMN_0195 |
| 3.45 | truncated triacylglycerol lipase precursor | 5330103 | NWMN_0262 |
| 2.23 | hypothetical protein | 5330127 | NWMN_0288 |
| 2.23 | phage terminase large subunit | 5330128 | NWMN_0289 |
| 2.23 | phage portal protein | 5330129 | NWMN_0290 |
| 2.23 | hypothetical protein | 5330130 | NWMN_0291 |
| 5.01 | phage major head protein | 5330133 | NWMN_0294 |
| 4.08 | hypothetical protein | 5330134 | NWMN_0295 |
| 3.27 | hypothetical protein | 5330135 | NWMN_0296 |
| 3.27 | hypothetical protein | 5330136 | NWMN_0297 |
| 6.05 | hypothetical protein | 5330137 | NWMN_0298 |
| 4.27 | hypothetical protein | 5330138 | NWMN_0299 |
| 5.96 | hypothetical protein | 5330139 | NWMN_0300 |
| 2.91 | phage tape measure protein | 5330141 | NWMN_0302 |
| 2.64 | hypothetical protein | 5330144 | NWMN_0305 |
| 2.64 | hypothetical protein | 5330145 | NWMN_0306 |
| 2.64 | hypothetical protein | 5330146 | NWMN_0307 |
| 2.87 | hypothetical protein | 5330147 | NWMN_0308 |
| 2.21 | amidase | 5330152 | NWMN_0313 |
| 3.37 | hypothetical protein | 5330167 | NWMN_0328 |
| 13.56 | hypothetical protein | 5330196 | NWMN_0362 |
| 2.76 | superantigen-like protein | 5332019 | NWMN_0400 |
| 2.32 | hypothetical protein | 5332475 | NWMN_0401 |
| 30.38 | hypothetical protein | 5330217 | NWMN_0402 |
| 2.18 | glutamate synthase subunit beta | 5331962 | NWMN_0437 |
| 20.14 | sensor histidine kinase SaeS | 5330385 | NWMN_0674 |
| 20.14 | DNA-binding response regulator SaeR | 5332432 | NWMN_0675 |
| 20.14 | hypothetical protein | 5332431 | NWMN_0676 |
| 36.14 | hypothetical protein | 5330386 | NWMN_0677 |
| 7.46 | extracellular matrix and plasma binding protein | 5330439 | NWMN_0758 |
| 8.63 | thermonuclease precursor | 5330440 | NWMN_0760 |
| 2.45 | hypothetical protein | 5330448 | NWMN_0769 |
| 16.79 | hypothetical protein | 5330653 | NWMN_1066 |
| 20.01 | formyl peptide receptor-like 1 inhibitory protein | 5330654 | NWMN_1067 |
| 24.77 | hypothetical protein | 5330656 | NWMN_1069 |
| 27.58 | hypothetical protein | 5330657 | NWMN_1070 |
| 3.75 | superantigen-like protein | 5330663 | NWMN_1077 |
| 4.94 | DNA-binding protein | 5331890 | NWMN_1083 |
| 5.1 | anti protein | 5331661 | NWMN_1084 |
| 2.56 | homoserine dehydrogenase | 5332539 | NWMN_1240 |
| 2.35 | indole-3-glycerol-phosphate synthase | 5332564 | NWMN_1282 |
| 2.35 | N-(5-phosphoribosyl) anthranilate isomerase | 5332563 | NWMN_1283 |
| 2.35 | tryptophan synthase subunit beta | 5332566 | NWMN_1284 |
| 2.35 | tryptophan synthase subunit alpha | 5332562 | NWMN_1285 |
| 12.47 | alanine dehydrogenase | 5332036 | NWMN_1349 |
| 2.08 | hypothetical protein | 5330856 | NWMN_1442 |
| 2.2 | threonyl-tRNA synthetase | 5332109 | NWMN_1576 |
| 2.1 | hypothetical protein | 5332472 | NWMN_1618 |
| 3.08 | serine protease SplA | 5332495 | NWMN_1706 |
| 2.54 | phage holin | 5331057 | NWMN_1770 |
| 2.74 | phage major tail protein | 5331070 | NWMN_1783 |
| 2.61 | hypothetical protein | 5331075 | NWMN_1788 |
| 2.14 | phage head protein | 5331076 | NWMN_1789 |
| 4.31 | phage endodeoxyribonuclease | 5331090 | NWMN_1803 |
| 4.07 | hypothetical protein pmtA | 5331135 | NWMN_1866 |
| 4.07 | ABC transporter ATP-binding protein | 5331136 | NWMN_1867 |
| 4.07 | hypothetical protein | 5331137 | NWMN_1868 |
| 3.38 | ABC transporter ATP-binding protein | 5331138 | NWMN_1869 |
| 3.38 | GntR family regulatory protein | 5331139 | NWMN_1870 |
| 53.79 | MHC class II analog protein | 5331141 | NWMN_1872 |
| 29.8 | truncated beta-hemolysin | 5332118 | NWMN_1873 |
| 5.32 | hypothetical protein | 5331142 | NWMN_1874 |
| 5.32 | hypothetical protein | 5331143 | NWMN_1875 |
| 24.27 | complement inhibitor SCIN | 5331144 | NWMN_1876 |
| 3.38 | leukocidin/hemolysin toxin subunit F | 5331190 | NWMN_1927 |
| 2.19 | hypothetical protein | 5332465 | NWMN_1941 |
| 2.24 | isopropylmalate isomerase small subunit | 5332079 | NWMN_1966 |
| 11.62 | immunoglobulin G-binding protein Sbi | 5331420 | NWMN_2317 |
| 62.41 | gamma-hemolysin component A | 5332443 | NWMN_2318 |
| 20.16 | gamma-hemolysin component C | 5332008 | NWMN_2319 |
| 14.71 | gamma hemolysin, component B | 5332010 | NWMN_2320 |
| 7.38 | hypothetical protein | 5332009 | NWMN_2321 |
| 2.69 | lipase precursor | 5332031 | NWMN_2569 |
| 3.03 | pyrrolidone-carboxylate peptidase | 5331611 | NWMN_2588 |
