# Supplementary material for: SaeRS-Dependent Inhibition of Biofilm Formation in Staphylococcus aureus Newman
Source: PLoS One. 2015 Apr 8;10(4):e0123027. doi: 10.1371/journal.pone.0123027 (PMC4390220; doi:10.1371/journal.pone.0123027)
Supplement: S5 Table — (DOCX) [file pone.0123027.s009.docx]

**Table S5. Genes up regulated in CYL11771 (*ΔsaeRS*) relative to wild type Newman (CYL5876).**

| **Fold-change** | **Gene Name or annotation** | **NCBI ID** | **Locus tag** |
| --- | --- | --- | --- |
| 4.46 | N-acetylmuramoyl-L-alanine amidase AAA precursor | 5330231 | NWMN_0429 |
| 4.36 | sorbitol dehydrogenase | 5330037 | NWMN_0183 |
| 4.35 | urease subunit gamma | 5331322 | NWMN_2188 |
| 4.08 | L-serine dehydratase, iron-sulfur-dependent, alpha subunit | 5331501 | NWMN_2429 |
| 4.03 | hydroxyethylthiazole kinase | 5332536 | NWMN_1996 |
| 4.03 | transcriptional regulator TenA family protein | 5332535 | NWMN_1998 |
| 4.03 | phosphomethylpyrimidine kinase | 5332538 | NWMN_1997 |
| 3.89 | urease accessory protein UreF | 5332585 | NWMN_2192 |
| 3.89 | urease accessory protein UreE | 5332583 | NWMN_2191 |
| 3.86 | urease subunit alpha | 5332582 | NWMN_2190 |
| 3.86 | urease subunit beta | 5332581 | NWMN_2189 |
| 3.7 | thiamine-phosphate pyrophosphorylase | 5332232 | NWMN_1995 |
| 3.6 | L-serine dehydratase, iron-sulfur-dependent, beta subunit | 5332458 | NWMN_2430 |
| 3.53 | urease accessory protein UreD | 5332587 | NWMN_2194 |
| 3.53 | urease accessory protein UreG | 5332586 | NWMN_2193 |
| 3.52 | capsular polysaccharide biosynthesis protein glycosyltransferase CapL | 5331805 | NWMN_0106 |
| 3.38 | ABC transporter ATP-binding protein | 5331421 | NWMN_2328 |
| 3.32 | capsular polysaccharide biosynthesis protein CapP | 5331809 | NWMN_0110 |
| 3.27 | glycerophosphoryl diester phosphodiesterase | 5330489 | NWMN_0830 |
| 3.24 | hypothetical protein | 5330563 | NWMN_0945 |
| 3.23 | hypothetical protein | 5332303 | NWMN_0943 |
| 3.23 | hypothetical protein | 5330562 | NWMN_0944 |
| 3.19 | capsular polysaccharide biosynthesis protein CapM | 5331806 | NWMN_0107 |
| 3.19 | capsular polysaccharide biosynthesis protein CapN | 5331807 | NWMN_0108 |
| 3.13 | capsular polysaccharide biosynthesis protein CapK | 5331804 | NWMN_0105 |
| 3.07 | phosphoribosylamine--glycine ligase | 5332306 | NWMN_0942 |
| 3.04 | phosphoribosylglycinamide formyltransferase | 5332309 | NWMN_0940 |
| 3 | capsular polysaccharide biosynthesis protein CapO | 5331808 | NWMN_0109 |
| 3 | capsular polysaccharide synthesis enzyme O-acetyl transferase CapH | 5331801 | NWMN_0102 |
| 2.96 | bifunctional phosphoribosylaminoimidazolecarboxamide formyltransferase/IMP cyclohydrolase | 5332310 | NWMN_0941 |
| 2.95 | hypothetical protein | 5330089 | NWMN_0246 |
| 2.95 | hypothetical protein | 5330827 | NWMN_1396 |
| 2.9 | phosphoribosylaminoimidazole-succinocarboxamide synthase | 5332307 | NWMN_0935 |
| 2.84 | acetyl-CoA carboxylase, biotin carboxyl carrier protein | 5331709 | NWMN_1508 |
| 2.81 | capsular polysaccharide biosynthesis protein CapJ | 5331803 | NWMN_0104 |
| 2.79 | acetyl-CoA biotin carboxylase | 5330899 | NWMN_1507 |
| 2.79 | LamB/YcsF family protein | 5330898 | NWMN_1506 |
| 2.77 | 30S ribosomal protein S17 | 5332377 | NWMN_2143 |
| 2.63 | hypothetical protein | 5330828 | NWMN_1397 |
| 2.63 | hypothetical protein | 5330829 | NWMN_1398 |
| 2.58 | capsular polysaccharide synthesis enzyme CapG | 5331800 | NWMN_0101 |
| 2.58 | hypothetical protein | 5330897 | NWMN_1505 |
| 2.51 | Drp35 | 5331610 | NWMN_2586 |
| 2.5 | hypothetical protein | 5330900 | NWMN_1510 |
| 2.5 | hypothetical protein | 5331707 | NWMN_1509 |
| 2.47 | queuine tRNA-ribosyltransferase | 5330921 | NWMN_1541 |
| 2.47 | flavohemoprotein | 5330030 | NWMN_0175 |
| 2.46 | tRNA | 5331698 | NWMN_tRNA53 |
| 2.43 | 30S ribosomal protein S8 | 5332371 | NWMN_2138 |
| 2.42 | hypothetical protein | 5330974 | NWMN_1650 |
| 2.39 | capsular polysaccharide synthesis enzyme CapF | 5331799 | NWMN_0100 |
| 2.35 | 2-oxoglutarate ferredoxin oxidoreductase subunit beta | 5332280 | NWMN_1199 |
| 2.33 | di-/tripeptide ABC transporter | 5330400 | NWMN_0696 |
| 2.33 | 50S ribosomal protein L6 | 5332381 | NWMN_2137 |
| 2.32 | 50S ribosomal protein L18 | 5332408 | NWMN_2136 |
| 2.28 | hypothetical protein | 5331363 | NWMN_2244 |
| 2.25 | NAD dependent epimerase/dehydratase family protein | 5331434 | NWMN_2341 |
| 2.19 | AtlA, bifunctional autolysin precursor | 5330556 | NWMN_0922 |
| 2.16 | spermidine/putrescine ABC transporter ATP-binding protein | 5330576 | NWMN_0965 |
| 2.16 | spermidine/putrescine ABC transporter permease protein | 5332282 | NWMN_0966 |
| 2.15 | hypothetical protein | 5330310 | NWMN_0570 |
| 2.12 | putative transposase | 5331338 | NWMN_2214 |
| 2.11 | hypothetical protein | 5329932 | NWMN_0027 |
| 2.08 | hypothetical protein | 5332119 | NWMN_1827 |
| 2.03 | undecaprenyldiphospho-muramoylpentapeptide beta-N- acetylglucosaminyltransferase | 5330789 | NWMN_1330 |
| 2.02 | UDP-N-acetylmuramoyl-L-alanyl-D-glutamate synthetase | 5332163 | NWMN_1093 |
