# Supplementary material for: SaeRS-Dependent Inhibition of Biofilm Formation in Staphylococcus aureus Newman
Source: PLoS One. 2015 Apr 8;10(4):e0123027. doi: 10.1371/journal.pone.0123027 (PMC4390220; doi:10.1371/journal.pone.0123027)
Supplement: S6 Table — (DOCX) [file pone.0123027.s010.docx]

**Table S6. Genes up regulated in CYL11771 (*ΔsaeRS*) relative to CYL11481 (saeS^L^).**

| **Fold-change** | **Gene annotation** | **NCBI ID** | **Locus tag** |
| --- | --- | --- | --- |
| 5.44 | DNA-binding protein | 5331890 | NWMN_1083 |
| 5.15 | hypothetical protein | 5330119 | NWMN_0280 |
| 5.07 | tandem lipoprotein | 5332093 | NWMN_0408 |
| 4.32 | phage major head protein | 5330133 | NWMN_0294 |
| 3.98 | urease subunit gamma | 5331322 | NWMN_2188 |
| 3.5 | choline dehydrogenase | 5331560 | NWMN_2509 |
| 3.49 | glycine betaine aldehyde dehydrogenase | 5331561 | NWMN_2510 |
| 3.21 | hypothetical protein | 5331089 | NWMN_1802 |
| 3.21 | hypothetical protein | 5331087 | NWMN_1800 |
| 3.21 | hypothetical protein | 5331088 | NWMN_1801 |
| 3.12 | hypothetical protein | 5330138 | NWMN_0299 |
| 2.85 | endodeoxyribonuclease | 5330609 | NWMN_1005 |
| 2.75 | urease subunit alpha | 5332582 | NWMN_2190 |
| 2.75 | urease subunit beta | 5332581 | NWMN_2189 |
| 2.67 | hypothetical protein | 5331095 | NWMN_1808 |
| 2.52 | hypothetical protein | 5332336 | NWMN_0206 |
| 2.31 | Ser-Asp rich fibrinogen/bone sialoprotein-binding protein SdrE | 5332463 | NWMN_0525 |
| 2.28 | O-Acetyl serine sulfhydrylase | 5332487 | NWMN_0060 |
| 2.28 | ornithine cyclodeaminase | 5332444 | NWMN_0061 |
| 2.27 | urease accessory protein UreF | 5332585 | NWMN_2192 |
| 2.27 | urease accessory protein UreE | 5332583 | NWMN_2191 |
| 2.25 | anti protein | 5331661 | NWMN_1084 |
| 2.16 | hypothetical protein | 5331085 | NWMN_1798 |
| 2.16 | hypothetical protein | 5331084 | NWMN_1797 |
| 2.15 | choline transporter | 5331564 | NWMN_2513 |
| 2.15 | hypothetical protein | 5331363 | NWMN_2244 |
| 2.13 | phage exonuclease | 5331186 | NWMN_1922 |
| 2.13 | hypothetical protein | 5332597 | NWMN_0541 |
| 2.12 | precorrin-2 dehydrogenase | 5331568 | NWMN_2517 |
| 2.12 | hypothetical protein | 5331562 | NWMN_2511 |
| 2.08 | hypothetical protein | 5330089 | NWMN_0246 |
| 2.06 | di-/tripeptide ABC transporter | 5330400 | NWMN_0696 |
| 2.06 | putative transposase | 5331338 | NWMN_2214 |
| 2.02 | urease accessory protein UreD | 5332587 | NWMN_2194 |
| 2.02 | urease accessory protein UreG | 5332586 | NWMN_2193 |
