# Supplementary material for: SaeRS-Dependent Inhibition of Biofilm Formation in Staphylococcus aureus Newman
Source: PLoS One. 2015 Apr 8;10(4):e0123027. doi: 10.1371/journal.pone.0123027 (PMC4390220; doi:10.1371/journal.pone.0123027)
Supplement: S7 Table — (DOCX) [file pone.0123027.s011.docx]

**Table S7. Genes up regulated in CYL11481 (saeS^L^) relative to wild type Newman (CYL5876).**

| **Fold change** | **Gene annotation** | **NCBI ID** | **Locus tag** |
| --- | --- | --- | --- |
| 3.78 | acetyl-CoA acetyltransferase | 5330184 | NWMN_0346 |
| 3.59 | tandem lipoprotein | 5332092 | NWMN_0407 |
| 3.43 | ABC transporter ATP-binding protein | 5331421 | NWMN_2328 |
| 3.34 | 50S ribosomal protein L24 | 5332370 | NWMN_2141 |
| 3.26 | 50S ribosomal protein L18 | 5332408 | NWMN_2136 |
| 3.23 | 30S ribosomal protein S5 | 5332392 | NWMN_2135 |
| 3.23 | 50S ribosomal protein L22 | 5332406 | NWMN_2147 |
| 3.17 | 30S ribosomal protein S17 | 5332377 | NWMN_2143 |
| 3.13 | CTP synthetase | 5331237 | NWMN_2031 |
| 3.11 | 50S ribosomal protein L30 | 5332378 | NWMN_2134 |
| 3.03 | 30S ribosomal protein S8 | 5332371 | NWMN_2138 |
| 3.01 | 50S ribosomal protein L29 | 5332421 | NWMN_2144 |
| 3.01 | 50S ribosomal protein L16 | 5332391 | NWMN_2145 |
| 2.98 | L-serine dehydratase, iron-sulfur-dependent, alpha subunit | 5331501 | NWMN_2429 |
| 2.97 | glutamine synthetase repressor | 5330720 | NWMN_1216 |
| 2.95 | 50S ribosomal protein L6 | 5332381 | NWMN_2137 |
| 2.93 | glutamine synthetase | 5331956 | NWMN_1217 |
| 2.9 | hypothetical protein | 5332276 | NWMN_0081 |
| 2.89 | 50S ribosomal protein L5 | 5332418 | NWMN_2140 |
| 2.8 | tRNA | 5331689 | NWMN_tRNA44 |
| 2.79 | tRNA | 5331698 | NWMN_tRNA53 |
| 2.74 | capsular polysaccharide biosynthesis protein glycosyltransferase CapL | 5331805 | NWMN_0106 |
| 2.7 | hypothetical protein | 5331427 | NWMN_2334 |
| 2.65 | staphylokinase precursor (sak) | 5331146 | NWMN_1880 |
| 2.61 | capsular polysaccharide biosynthesis protein CapM | 5331806 | NWMN_0107 |
| 2.61 | capsular polysaccharide biosynthesis protein CapN | 5331807 | NWMN_0108 |
| 2.58 | tRNA | 5331680 | NWMN_tRNA35 |
| 2.52 | capsular polysaccharide biosynthesis protein CapP | 5331809 | NWMN_0110 |
| 2.46 | capsular polysaccharide biosynthesis protein CapO | 5331808 | NWMN_0109 |
| 2.42 | capsular polysaccharide biosynthesis protein CapK | 5331804 | NWMN_0105 |
| 2.39 | capsular polysaccharide biosynthesis protein CapJ | 5331803 | NWMN_0104 |
| 2.34 | 30S ribosomal protein S20 | 5330887 | NWMN_1488 |
| 2.25 | capsular polysaccharide synthesis enzyme O-acetyl transferase CapH | 5331801 | NWMN_0102 |
| 2.24 | capsular polysaccharide biosynthesis protein CapI | 5331802 | NWMN_0103 |
| 2.19 | alanine dehydrogenase | 5330941 | NWMN_1603 |
| 2.19 | PTS system, mannitol-specific IIBC component | 5331954 | NWMN_2057 |
| 2.18 | glycine cleavage system aminomethyltransferase T | 5331944 | NWMN_1441 |
| 2.13 | hypothetical protein | 5330897 | NWMN_1505 |
| 2.11 | 2-oxoisovalerate dehydrogenase, E2 component | 5330845 | NWMN_1421 |
| 2.04 | hypothetical protein | 5330067 | NWMN_0224 |
| 2.04 | signal recognition particle protein | 5330697 | NWMN_1147 |
| 2.04 | UDP-N-acetylmuramoyl-L-alanyl-D-glutamate synthetase | 5332163 | NWMN_1093 |
| 2 | cell division protein FtsQ | 5332166 | NWMN_1094 |
