# Supplementary material for: SaeRS-Dependent Inhibition of Biofilm Formation in Staphylococcus aureus Newman
Source: PLoS One. 2015 Apr 8;10(4):e0123027. doi: 10.1371/journal.pone.0123027 (PMC4390220; doi:10.1371/journal.pone.0123027)
Supplement: S8 Table — (DOCX) [file pone.0123027.s012.docx]

**Table S8. Newman genes exhibiting an apparent dose-dependent response to SaeRS levels.**

| **Fold** | **Decrease** |  |  |  |
| --- | --- | --- | --- | --- |
| **wt:*Δsa****eRS* | **wt:*saeS^L^*** | **Gene annotation** | **NCBI ID** | **Locus tag** |
| 978.01 | 16.79 | hypothetical protein | 5330653 | NWMN_1066 |
| 864.3 | 36.14 | hypothetical protein | 5330386 | NWMN_0677 |
| 852.35 | 24.77 | hypothetical protein | 5330656 | NWMN_1069 |
| 551.34 | 20.14 | sensor histidine kinase *saeS* | 5330385 | NWMN_0674 |
| 551.34 | 20.14 | DNA-binding response regulator *saeR* | 5332432 | NWMN_0675 |
| 551.34 | 20.14 | hypothetical protein | 5332431 | NWMN_0676 |
| 452.36 | 30.38 | hypothetical protein | 5330217 | NWMN_0402 |
| 409.52 | 27.58 | hypothetical protein | 5330657 | NWMN_1070 |
| 242.9 | 53.79 | MHC class II analog protein *map* | 5331141 | NWMN_1872 |
| 226.61 | 20.16 | gamma-hemolysin component C | 5332008 | NWMN_2319 |
| 145.03 | 5.44 | chp | 5332454 | NWMN_1877 |
| 127.03 | 62.41 | gamma-hemolysin component A | 5332443 | NWMN_2318 |
| 102.19 | 11.62 | immunoglobulin G-binding protein Sbi | 5331420 | NWMN_2317 |
| 98 | 24.27 | complement inhibitor (scn) | 5331144 | NWMN_1876 |
| 65.81 | 14.71 | gamma hemolysin, component B | 5332010 | NWMN_2320 |
| 46.51 | 12.47 | alanine dehydrogenase | 5332036 | NWMN_1349 |
| 41.13 | 3.38 | leukocidin/hemolysin toxin subunit F | 5331190 | NWMN_1927 |
| 37.1 | 8.63 | thermonuclease precursor *nuc* | 5330440 | NWMN_0760 |
| 28.22 | 13.56 | hypothetical protein | 5330196 | NWMN_0362 |
| 25.31 | 7.46 | extracellular matrix and plasma binding protein | 5330439 | NWMN_0758 |
| 20.36 | 20.01 | formyl peptide receptor-like 1 inhibitory protein | 5330654 | NWMN_1067 |
| 18.3 | 3.45 | truncated triacylglycerol lipase precursor | 5330103 | NWMN_0262 |
| 14.61 | 7.4 | staphylocoagulase precursor | 5330026 | NWMN_0166 |
| 9.88 | 5.32 | hypothetical protein | 5331142 | NWMN_1874 |
| 9.88 | 5.32 | hypothetical protein | 5331143 | NWMN_1875 |
| 7.37 | 3.75 | superantigen-like protein | 5330663 | NWMN_1077 |
| 5.26 | 3.37 | hypothetical protein | 5330167 | NWMN_0328 |
| 3.93 | 2.51 | hypothetical protein | 5329982 | NWMN_0112 |
| 3.52 | 2.19 | hypothetical protein | 5332465 | NWMN_1941 |
