# Supplementary material for: SaeRS-Dependent Inhibition of Biofilm Formation in Staphylococcus aureus Newman
Source: PLoS One. 2015 Apr 8;10(4):e0123027. doi: 10.1371/journal.pone.0123027 (PMC4390220; doi:10.1371/journal.pone.0123027)
Supplement: S9 Table — (DOCX) [file pone.0123027.s013.docx]

**Table S9. Bacteriophage genes up regulated by *saeS^P^*.**

| **Gene annotation** | **Fold decrease *saeS^L^*** | **Fold decrease *ΔsaeSR*** | **NCBI ID** | **Locus tag** |
| --- | --- | --- | --- | --- |
|  |  |  |  |  |
| **ΦNM 1:** |  |  |  |  |
| phage holin | 2.54 |  | 5331057 | NWMN_1770 |
| phage major tail protein | 2.74 |  | 5331070 | NWMN_1783 |
| hypothetical protein | 2.61 |  | 5331075 | NWMN_1788 |
| phage head protein | 2.14 |  | 5331076 | NWMN_1789 |
| phage endodeoxyribonuclease | 4.31 |  | 5331090 | NWMN_1803 |
|  |  |  |  |  |
| **ΦNM2:** |  |  |  |  |
| hypothetical protein |  | 2.08 | 5330606 | NWMN_1002 |
|  |  |  |  |  |
| **ΦNM3:** |  |  |  |  |
| hypothetical protein | 4.07 | 2.66 | 5331137 | NWMN_1868 |
| MHC class II analog protein | 53.8 | 242.9 | 5331141 | NWMN_1872 |
| truncated beta-hemolysin | 29.8 | 29.8 | 5332118 | NWMN_1873 |
| hypothetical protein | 5.32 | 9.88 | 5331142 | NWMN_1874 |
| hypothetical protein | 5.32 | 9.88 | 5331143 | NWMN_1875 |
| complement inhibitor (scn) | 24.3 | 98 | 5331144 | NWMN_1876 |
| chemotaxis-inhibiting protein (chp) | 5.4 | 145 | 5332454 | NWMN_1877 |
| hypothetical protein | 2.05 | 4.25 | 5331155 | NWMN_1891 |
| hypothetical protein | 2.05 | 4.25 | 5331156 | NWMN_1892 |
| phage head-tail adaptor | 2.05 | 4.25 | 5331157 | NWMN_1893 |
| hypothetical protein | 2.05 | 4.25 | 5331158 | NWMN_1894 |
| hypothetical protein | 2.05 | 4.25 | 5331159 | NWMN_1895 |
| phage major capsid protein |  | 4.26 | 5331160 | NWMN_1896 |
| phage Clp-like protease |  | 2.4 | 5331161 | NWMN_1897 |
| phage portal protein |  | 2.4 | 5331162 | NWMN_1898 |
| hypothetical protein |  | 2.91 | 5331177 | NWMN_1913 |
| hypothetical protein |  | 2.91 | 5331178 | NWMN_1914 |
| hypothetical protein |  | 2.91 | 5331179 | NWMN_1915 |
|  |  |  |  |  |
| **ΦNM4:** |  |  |  |  |
| integrase |  | 6.08 | 5331946 | NWMN_0263 |
| hypothetical protein | 2.23 |  | 5330127 | NWMN_0288 |
| phage terminase large subunit | 2.23 |  | 5330128 | NWMN_0289 |
| phage portal protein | 2.23 |  | 5330129 | NWMN_0290 |
| hypothetical protein | 2.23 |  | 5330130 | NWMN_0291 |
| phage major head protein | 5.01 |  | 5330133 | NWMN_0294 |
| hypothetical protein | 4.08 |  | 5330134 | NWMN_0295 |
| hypothetical protein | 3.27 |  | 5330135 | NWMN_0296 |
| hypothetical protein | 3.27 |  | 5330136 | NWMN_0297 |
| hypothetical protein | 6.05 |  | 5330137 | NWMN_0298 |
| hypothetical protein | 4.27 |  | 5330138 | NWMN_0299 |
| hypothetical protein | 5.96 |  | 5330139 | NWMN_0300 |
| phage tape measure protein | 2.91 |  | 5330141 | NWMN_0302 |
| hypothetical protein | 2.64 |  | 5330144 | NWMN_0305 |
| hypothetical protein | 2.64 |  | 5330145 | NWMN_0306 |
| hypothetical protein | 2.64 |  | 5330146 | NWMN_0307 |
| hypothetical protein | 2.87 |  | 5330147 | NWMN_0308 |
| amidase | 2.21 |  | 5330152 | NWMN_0313 |
