# Supplementary material for: SaeRS-Dependent Inhibition of Biofilm Formation in Staphylococcus aureus Newman
Source: PLoS One. 2015 Apr 8;10(4):e0123027. doi: 10.1371/journal.pone.0123027 (PMC4390220; doi:10.1371/journal.pone.0123027)
Supplement: S10 Table — (DOCX) [file pone.0123027.s014.docx]

**Table S10. Oligonucleotide primers used for real-time PCR.**

| **Primer** | **Sequence** |
| --- | --- |
| atlA F | GGTTTCGACGGTGTTGTTGG |
| atlA R | TTATCTTGGGGTGTCGGTGC |
| SGATLF | ACAGCTAGAACTTCTCCGTATTT |
| SGATLR | GTTGACTTGTGATGTACCCTTTC |
| lrgA set 4 forward | GCCAATTCCTATGCCTGGATCAGT |
| lrgA set 4 reverse | TCGACTTCGCCTAACTTAACAGCACC |
| lytS 1 | TTGTAGTCATCCGAATTGCCCGCTTAGAGCA |
| lytS 2 | AACTTACTTTGCGTTTCGGCTTCACCAAGTTCAA |
| NWMN_0429 F | ATGCGGCTACAACTCACACA |
| NWMN_0429 R | ATTGCTCGCAGCGTTACTTG |
| sgarlR1 | TTACGGTGCAGACGATTATATAG |
| sgarlR2 | TACCGTTGACATCGATAATATCC |
| SGhu 2 | ATCCAAAACTCACTTGCTAAAGG |
| SGhu 3 | ACCAGCTTTGAATGCTGGAAC |
| SGSae R3 | CTGATCGTGGATGATGAACAA |
| SGSae R4 | CTTCTTTACCGCTAGTTGTCG |
